# Different traditional Chinese medicine injections combined with conventional therapy for sepsis-induced myocardial dysfunction: a systematic review and network meta-analysis

**DOI:** 10.3389/fphar.2025.1688113

**Published:** 2026-01-05

**Authors:** Wenjie Deng, Xiaolei Fang, Po Huang

**Affiliations:** 1 Beijing University of Traditional Chinese Medicine, Beijing, China; 2 Dongfang Hospital, Beijing University of Traditional Chinese Medicine, Beijing, China

**Keywords:** traditional Chinese medicine injections, sepsis-induced myocardial dysfunction, systematic review, network meta-analysis, randomized controlled trial

## Abstract

**Objective:**

As the use of Traditional Chinese Medicine injections (TCMIs) for sepsis-induced myocardial dysfunction (SIMD) becomes increasingly diverse, this study aims to evaluate their efficacy, optimal combinations, and safety.

**Methods:**

A comprehensive computerized search was conducted across multiple databases, including PubMed, Web of Science, the Cochrane Library, China National Knowledge Infrastructure, VIP Database, Wanfang Database, and SinoMed, for randomized controlled trials (RCTs) on TCMIs for treating SIMD, covering the period from the inception of these databases to 25 June 2025. The methodological quality of the included studies was assessed using the Cochrane Risk of Bias tool (ROB 2.0). A network meta-analysis was performed using Stata 18.0 software. The study protocol has been registered with PROSPERO.

**Results:**

A total of 31 randomized controlled trials (RCTs) were included, comprising 2,166 participants (intervention group: n = 1,099; control group: n = 1,067). Nine TCMIs were investigated: Shenmai injection (SM), Danshen Chuanxiongqin injection (DSCXQ), Xuebijing injection (XBJ), Shenfu injection (SF), Shuxuening injection (SXN), Xinmailong injection (XML), Huangqi injection (HQ), Danhong injection (DH), and Shenqi fuzheng injection (SQFZ). Based on the surface under the cumulative ranking curve values, (1) In reducing 28-day mortality, Shuxuening Injection combined with conventional therapy ranked first based on the Surface Under the Cumulative Ranking Curve (SUCRA) (relative risk [RR], 3.67; 95% confidence interval [CI], 1.09–12.32). (2) For the improvement of cardiac troponin I (cTnI) levels, Danshen Chuanxiongqin Injection combined with conventional therapy ranked first based on SUCRA (mean difference [MD], 0.87; 95% CI, 0.22–1.52). (3) In terms of left ventricular ejection fraction (LVEF) improvement, Shenqi Fuzheng injection combined with conventional therapy ranked first based on SUCRA (MD, −8.79; 95% CI, −15.99 to −1.59). (4) For the reduction of B-type natriuretic peptide (BNP), Xinmailong injection combined with conventional therapy ranked first based on SUCRA (MD, 353.37; 95% CI, 188.55–518.19). (5) Regarding the improvement of N-terminal pro-BNP (NT-proBNP), Danshen Chuanxiongqin injection combined with conventional therapy ranked first based on SUCRA (MD, 557.45; 95% CI, 177.68–937.22). In addition, no significant adverse reactions were reported in the relevant studies on the Shenmai, Danshen Chuanxiongqin, Shenfu, Shuxuening, Huangqi, Danhong and Shenqi fuzheng injections, whereas the Xuebijing and Xinmailong injections demonstrated varying degrees of adverse reactions.

**Conclusion:**

Compared to conventional therapy alone, the addition of TCMIs in the treatment of SIMD may confer advantages in reducing 28-day mortality, improving levels of cTnI, BNP, and NT-proBNP, as well as increasing LVEF levels. However, due to the low methodological quality of the studies—particularly in blinding and allocation concealment—and poorly documented composition and safety profiles of TCMIs, the reliability of these findings is compromised.

**Systematic Review Registration:**

https://www.crd.york.ac.uk/prospero/.

## 1 Introduction

Sepsis is a life-threatening organ dysfunction resulting from an unregulated host response (Srzić et al., 2022). The heart, as the most vulnerable target organ, exhibits significantly elevated mortality rates when complicated by cardiac dysfunction (Vieillard-Baron et al., 2008). Sepsis-induced myocardial dysfunction (SIMD) is characterized by intrinsic systolic and diastolic dysfunction of both the left and right sides of the heart (Lv and Wang, 2016). Current management strategies for SIMD primarily involve supportive care, including antimicrobials and fluid resuscitation. The pathogenesis of SIMD encompasses inflammatory activation, mitochondrial dysfunction, cardiomyocyte death, and microcirculatory impairment (Aissaoui et al., 2025). Consequently, early myocardial protection is crucial for improving outcomes in sepsis. TCMIs, which contain refined herbal bioactives, demonstrate multi-target efficacy in treating SIMD through metabolic enhancement, cytoprotection, and hemodynamic stabilization (Li M. et al., 2019; Chai et al., 2019). To evaluate the efficacy of various TCMIs for improving cardiac function in SIMD, this network meta-analysis systematically assesses nine clinically utilized TCMIs, providing evidence to assist clinicians in selecting the optimal therapeutic strategy.

## 2 Materials and methods

This Network Meta-Analysis (NMA) was prospectively registered with the International Prospective Register of Systematic Reviews (PROSPERO) under registration number CRD420251102252. Furthermore, this study was conducted in accordance with the Preferred Reporting Items for Systematic Reviews and Meta-Analyses (PRISMA) 2020 guidelines (Page et al., 2021) and the Cochrane Handbook for Systematic Reviews of Interventions (Higgins et al., 2011). Details are provided in Supplementary Appendix Table 1.

### 2.1 Standard evaluation of TCMIs

We standardized the scientific nomenclature of the botanical drug components on October 23 with reference to the MPNS (http://mpns.kew.org/mpns-portal/). The components of each TCMIs along with their standardized names are presented in Supplementary Appendix Table 2. Furthermore, these injections have been approved by the National Medical Products Administration of China and are widely utilized in clinical practice. Additional information on the Chinese medicine injections included in this study was sourced from a Chinese medical information platform, as detailed in Supplementary Appendix Table 3.

### 2.2 Eligibility criteria

#### 2.2.1 Study type: RCTs

#### 2.2.2 Study subjects

(1) patients ≥18 years diagnosed with sepsis or septic shock (Singer et al., 2016); (2) Meeting the diagnostic criteria for myocardial injury (Chinese Association of Integrative Medicine Critical Care Committee and Chinese Medical Doctor Association Cardiology Intervention Committee, 2022): Echocardiographic evidence: LVEF≤50%; Biomarker elevation: Elevated levels of cTnI, BNP and/or NT-ProBNP, CK-MB, suggesting myocardial injury or cardiac dysfunction.

#### 2.2.3 Interventions

All patients in both groups received conventional therapy according to established guidelines (Evans et al., 2021). This conventional therapy included early volume resuscitation, judicious use of vasoactive agents, broad-spectrum antibiotic medications, and organ function support as needed. Patients in the treatment group were administered additional TCMIs, with no restrictions imposed on the injection methods, dosages, or treatment duration.

#### 2.2.4 Outcome measures

The primary outcome was 28-day mortality, with secondary outcomes including: cTnI, BNP, NT-proBNP, LVEF.

### 2.3 Exclusion criteria

(1) Duplicate publications. (2) Studies lacking outcome reporting. (3) Unavailable data with failed author contact.

### 2.4 Search strategy

We conducted a systematic search of multiple databases, including PubMed, Web of Science, the Cochrane Library, China National Knowledge Infrastructure, VIP Database, Wanfang Database, and SinoMed, to identify RCTs evaluating the efficacy of TCMIs in treating sepsis-induced myocardial dysfunction (SIMD) from the inception of these databases until 25 June 2025. The search utilized a combination of subject headings and keywords, specifically: “Sepsis,” “myocardial injury,” “myocardial dysfunction,” “traditional Chinese medicine,” and “randomized controlled trial,” tailored to the specific characteristics of each database.

### 2.5 Study selection and data extraction

Two researchers independently conducted the literature screening process. Two researchers first removed duplicate records using EndNote, followed by screening title and abstract based on inclusion and exclusion criteria. Subsequently, Two researchers obtained full-text articles of the remaining publications. Through discussion and consensus, finalized the list of included studies and developed a standardized data extraction form to document key elements, such as (i) author aspects: first author and year of publication; (ii) patient aspects: sample size of each group (number of people in the intervention group, number of people in the control group), mean age, treatment, adverse effects, and outcome measures. Any disagreements during this process were resolved through consultation with a third researcher.

### 2.6 Risk of bias assessment

The methodological quality of the included studies was assessed using the Cochrane Risk of Bias Tool (ROB 2.0) recommended in the Cochrane Handbook for Systematic Reviews (Version 5.3). Two independent reviewers evaluated the following domains: randomization process, deviations from intended interventions, missing outcome data, measurement of the outcome and selection of the reported result. Each domain was rated as “low risk“ or “high risk“ or “unclear risk.“ The reviewers cross-checked their assessments, and any discrepancies were resolved through discussion. Finally, a quality assessment table was generated to summarize the risk of bias for each study.

### 2.7 Statistical analysis

Data management was conducted using Microsoft Excel and EndNote 20. Network meta-analysis was performed utilizing Stata software version 18.0. Network plots were generated to visualize the relationships between interventions. Closed loops in the evidence networks underwent inconsistency testing; otherwise, consistency models were applied. Effect measures were reported as relative risks (RRs) for dichotomous outcomes and mean differences (MDs) for continuous outcomes, both accompanied by 95% confidence intervals (CIs). RR and MD were calculated with the control group as the reference. Statistical significance was defined as 95% CIs excluding the null value (1 for RRs; 0 for MDs). Treatment rankings were evaluated using the surface under the cumulative ranking curve (SUCRA), where higher values indicated superior efficacy. The Confidence in Network Meta-Analysis (CINeMA) framework was employed to assess the certainty of evidence across six domains: within-study bias, reporting bias, indirectness, imprecision, heterogeneity, and incoherence. The CINeMA evaluation website is available at (https://cinema.ispm.unibe.ch/). Publication bias was assessed by generating comparison-adjusted funnel plots and conducting Egger’s test to identify potential reporting bias for primary outcomes. Finally, We explored sources of heterogeneity (I^2^ > 50% or p < 0.1) via subgroup analyses and assessed the robustness of the findings using sensitivity analyses.

## 3 Results

### 3.1 Study selection

**FIGURE 1:**
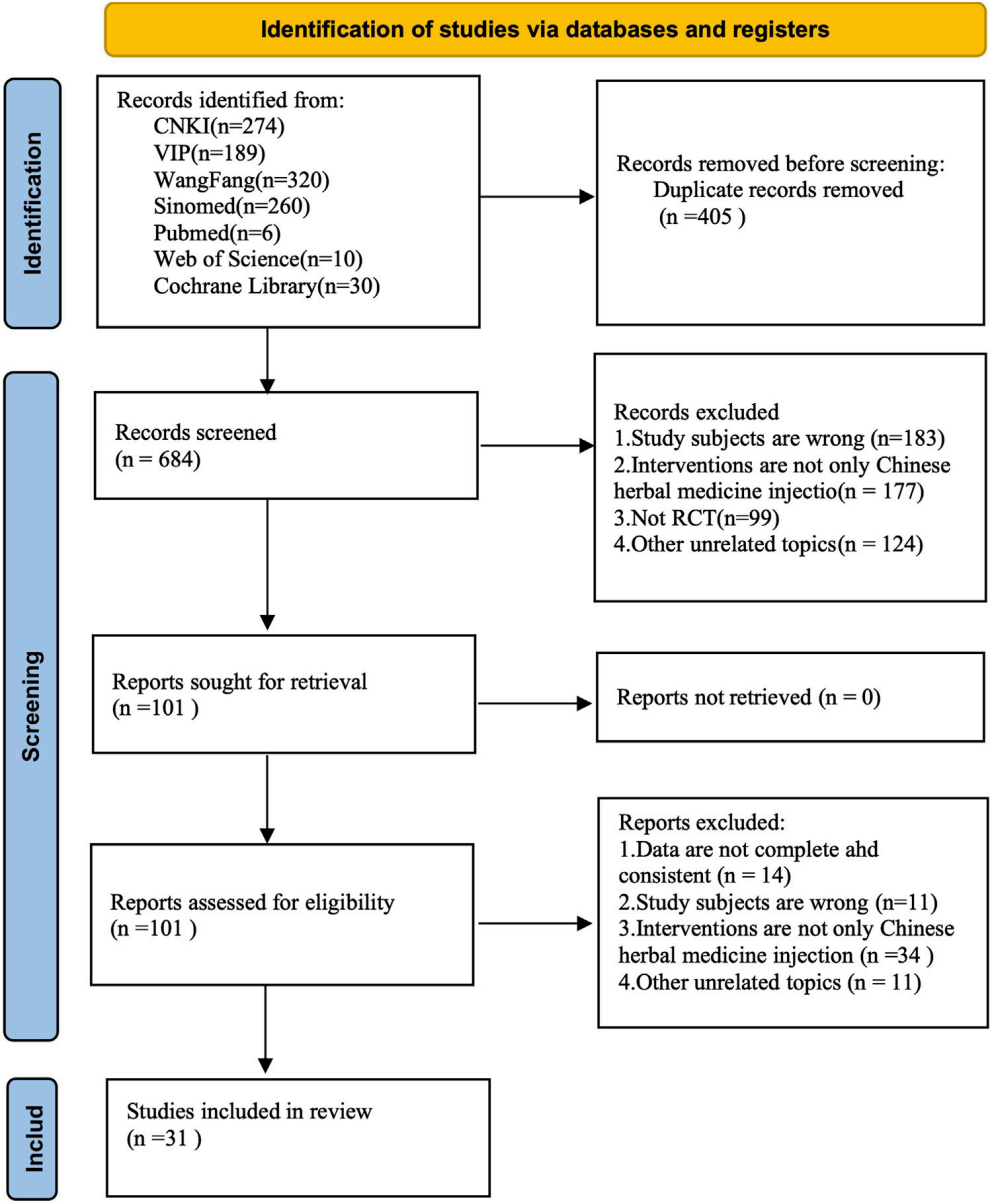
The PRISMA flow diagram of the study selection.

A total of 1,089 potentially relevant articles were identified. After removing 405 duplicates, 684 records underwent title/abstract screening, with 583 excluded. Full-text review of the remaining 101 articles led to the exclusion of 70 studies. Through this sequential screening process, 31 RCTs were ultimately included. The study selection flow is presented in Figure 1.

### 3.2 Study characteristics

A total of 31 RCTs were included in this analysis, comprising 2,166 participants (intervention group: n = 1,099; control group: n = 1,067). Nine TCMIs were investigated: Shenmai injection (SM), Danshen Chuanxiongqin injection (DSCXQ), Xuebijing injection (XBJ), Shenfu injection (SF), Shuxuening injection (SXN), Xinmailong injection (XML), Huangqi injection (HQ), Danhong injection (DH), and Shenqi Fuzheng injection (SQFZ). The baseline characteristics of the included studies are presented in Table 1.

**TABLE 1:** The baseline characteristics of the included studies.

Study	Sample size	Age T/C (mean or range years)	Intervention vs. control	Course	Outcomes
Chen et al. (2017)	T: 14 C: 14	T.57.5 ± 16.6 C.55.8 ± 12.3	SM + CT vs. CT	7 d	①②④
He et al. (2019)	T: 16 C: 16	T.61.19 ± 8.26 C.59.75 ± 8.66	SM + CT vs. CT	7 d	③
Yan et al. (2014)	T: 60 C: 63	T.65 ± 21 C.67 ± 23	SM + CT vs. CT	14 d	①⑤
Liu et al. (2018)	T: 53 C: 53	T.66.73 ± 4.80 C.67.04 ± 4.75	SM + CT vs. CT	7 d	①②⑤
He et al. (2016)	T: 57 C: 57	T.62.46 ± 6.3 C.59.32 ± 5.41	DSCXQ + CT vs. CT	14 d	①②③⑤
Wu et al. (2022)	T: 32 C: 31	T.72.53 ± 13.44 C.74.94 ± 12.20	XBJ + CT vs. CT	7 d	②
Feng et al. (2020)	T: 30 C: 30	T.49.2 ± 9.7 C.48.1 ± 10.4	XBJ + CT vs. CT	7 d	①②
Chen et al. (2019)	T: 30 C: 30	T.56.28 ± 2.62 C.58.28 ± 2.14	XBJ + CT vs. CT	7 d	①②⑤
Liang et al. (2010)	T: 14 C: 13	T. 49.9 ± 10.62 C.49.78 ± 12.56	XBJ + CT vs. CT	7 d	①
Han et al. (2020)	T: 30 C: 30	NA	XBJ + CTvs.CT	7 d	①④
Liu et al. (2014)	T: 45 C: 27	T.59.6 ± 16.1 C.57.2 ± 14.7	SF + CT vs. CT	7 d	②③⑤
Lei (2024)	T: 35 C: 35	T.68.73 ± 10.29 C.67.38 ± 13.06	SF + CT vs. CT	5 d	②③
Zhang and Li (2025)	T: 41 C: 41	NA	SF + CT vs. CT	3 d	②⑤
Xu et al. (2023)	T: 19 C: 20	T.63.33 ± 6.12 C.64.8 ± 7.47	SF + CT vs. CT	7 d	①⑤
Li et al. (2022)	T: 30 C: 19	T.73.74 ± 12.97 C.69.95 ± 11.28	SF + CT vs. CT	5 d	⑤
Lin et al. (2014)	T.30 C.30	T.57.8 ± 11.7 C. 59.8 ± 13.9	SF + CT vs. CT	14 d	⑤
Shen and Zhang (2017)	T: 30 C: 30	NA	SF + CT vs. CT	14 d	③④
Luo et al. (2018)	T: 30 C: 30	T.43.35 ± 4.31 C.41.20 ± 4.34	SF + CT vs. CT	7 d	①②④
Hu et al. (2016)	T: 32 C: 32	T.40.80 ± 2.70 C. 41.20 ± 3.80	SF + CT vs. CT	NA	①④
Li et al. (2019b)	T: 30 C: 30	T.19–59 C.18–60	XML + CT vs. CT	5 d	①②④
Han et al. (2017)	T: 32 C: 32	NA	XML + CT vs. CT	5 d	①②④
Luo et al. (2019)	T: 30 C: 30	NA	XML + CT vs. CT	7 d	①②④
Wang (2017)	T: 17 C: 19	T.54.65 ± 12.3 C.53.25 ± 11.87	XML + CT vs. CT	5 d	①②④
Zhang et al. (2018)	T: 27 C: 24	T.63.75 ± 12.24 C.60.56 ± 14.32	XML + CT vs. CT	5 d	①④
Zhu et al. (2019)	T: 23 C: 20	T.81.48 ± 6.466 C.75.7 ± 12.175	XML + CT vs. CT	5 d	②③④
He et al. (2021)	T: 96 C: 96	T.68.5 ± 13.2 C.70.1 ± 14.1	XML + CT vs. CT	NA	①②③④
Huang (2015)	T: 32 C: 32	T.63.1 ± 8.9 C.62.7 ± 8.6	HQ + CT vs. CT	14 d	①②
Xia (2019)	T: 48 C: 48	T.56.93 ± 4.07 C.56.94 ± 4.03	SXN + CT vs. CT	14 d	①③
Qi (2017)	T: 43 C: 43	T.64 ± 7 C.66 ± 8	SXN + CT vs. CT	14 d	①
Tao (2017)	T: 40 C: 40	NA	DH + CT vs. CT	7 d	①④
Wang (2019)	T: 49 C: 49	T.56.78 ± 17.34 C.64.63 ± 13.90	SQFZ + CT vs. CT	7 d	②③

CT, conventional therapy; SM, Shenmai injection; DSCX, Danshen Chuanxiongqin injection; XBJ, Xuebijing injection; SF, Shenfu injection; XML, Xinmailong injection; HQ, Huangqi injection; SXN, Shuxuening injection; DH, Danhong injection; SQFZ, Shenqifuzheng injection.

① cTn I; ② LVEF; ③ 28-day mortality; ④ BNP; ⑤ NT-proBNP.

### 3.3 Risk of bias of included studies

**FIGURE 2:**
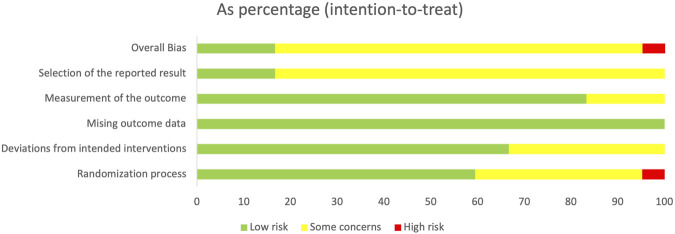
The risk of bias charts for the included.

The methodological quality of the 31 included studies was assessed using the Cochrane Risk of Bias 2 (ROB-2) tool. All studies reported randomization, with 16 studies (Chen et al., 2017; He et al., 2019; Yan et al., 2014; Liu et al., 2018; He et al., 2016; Wu et al., 2022; Feng et al., 2020; Chen et al., 2019; Liang et al., 2010; Han et al., 2020; Liu et al., 2014; Lei, 2024; Zhang and Li, 2025; Xu et al., 2023; Li et al., 2022; Shen and Zhang, 2017) using a random number table and 1 (Shen and Zhang, 2017) employing sealed envelopes, both rated as low risk. Twelve studies (Luo et al., 2018; Hu et al., 2016; Li X. et al., 2019; Han et al., 2017; Luo et al., 2019; Wang, 2017; Zhang et al., 2018; Zhu et al., 2019; He et al., 2021; Huang, 2015; Xia, 2019) mentioned randomization without specifying the method and were rated as moderate risk, while two studies (Qi, 2017; Tao, 2017) used admission sequence allocation and were rated as high risk. One study explicitly reported double-blinding (Feng et al., 2020). Regarding bias due to missing outcome data, all studies included predetermined outcome measures. There was no evidence of selective reporting, nor were any other discernible sources of bias detected. Overall, three studies were rated as low risk, 2 as high risk, and 26 as having some concerns. The risk of bias summary was presented in Figure 2. The risk of bias charts for the included studies was presented in Supplementary Appendix Table 4, while more detailed outcomes can be found in Supplementary Appendix Tables 5–9.

### 3.4 Primary outcome: 28-day mortality

#### 3.4.1 Network meta-analysis

**FIGURE 3:**
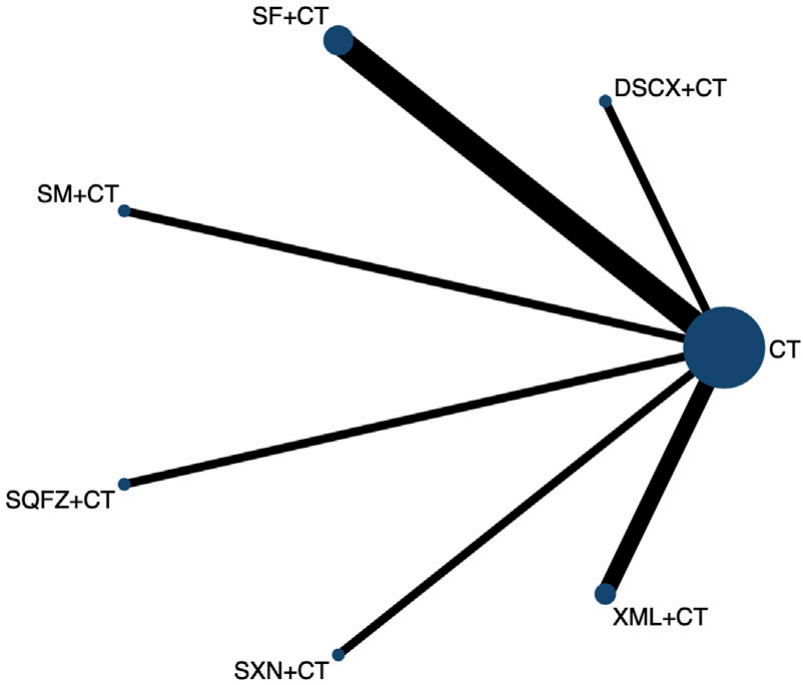
Evidence network diagram of 28-Day mortality of traditional Chinese medicine injection for treatment of SIMD. CT, conventional therapy; SM, Shenmai injection; DSCX, Danshen Chuanxiongqin injection; SF, Shenfu injection; XML, Xinmailong injection; SXN, Shuxuening injection; SQFZ, Shenqifuzheng injection. Each node represents an intervention, with node size proportional to the total sample size for each intervention. The width of connecting lines between nodes corresponds to the number of studies comparing each treatment pair.

**FIGURE 4:**
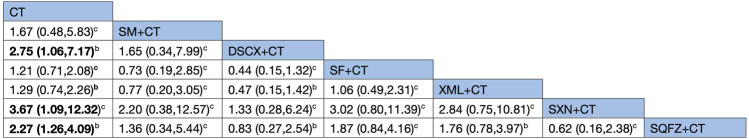
Net meta-analysis of 28-day mortality of traditional Chinese medicine injection for treatment of SIMD. ^a^Moderate quality of evidence. ^b^Low quality of evidence. ^c^Very low quality of evidence.

**FIGURE 5:**
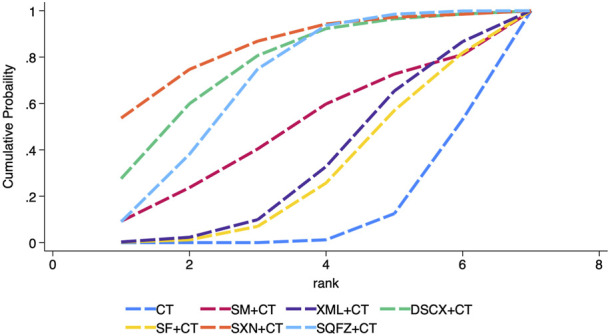
SUCRA ranking of 28-day mortality of traditional Chinese medicine injection for treatment of SIMD.

The 28-day mortality was reported in nine RCTs evaluating six TCMIs (Figure 3). The network meta-analysis demonstrated that DSCXQ combined with conventional therapy (CT) (2.75,95% CI: 1.06 to 7.17, low quality of evidence), SXN combined with CT (3.67, 95% CI: 1.09 to 12.32, very low quality of evidence), and SQFZ combined with CT (2.27,95% CI: 1.26 to 4.09, low quality of evidence) all showed statistically significant reductions in 28-day mortality compared to conventional therapy alone (P < 0.05) (Figure 4). The SUCRA-based treatment hierarchy was as follows: SXN + CT (84.3%) > DSCXQ + CT (76.0%) > SQFZ + CT (69.0%) > SM + CT (47.9%) > XML + CT (32.9%) > SF + CT (28.8%) (Figure 5).

#### 3.4.2 Confidence in evidence

The evidence confidence assessment utilizing the CINeMA framework rated the majority of primary outcomes as “low“ (12.9%) to “very low“ (87.1%), with a predominance of “very low“ ratings. Specifically, network estimates indicated low confidence for DSCXQ + CT and SQFZ + CT, while SXN + CT exhibited very low confidence, primarily due to imprecision and limitations in the studies. Although the evidence supporting SXN combinations remains clinically suggestive, the therapeutic implications should be interpreted with caution, given the overall low quality of the included studies.

#### 3.4.3 Publication bias analysis

**FIGURE 6:**
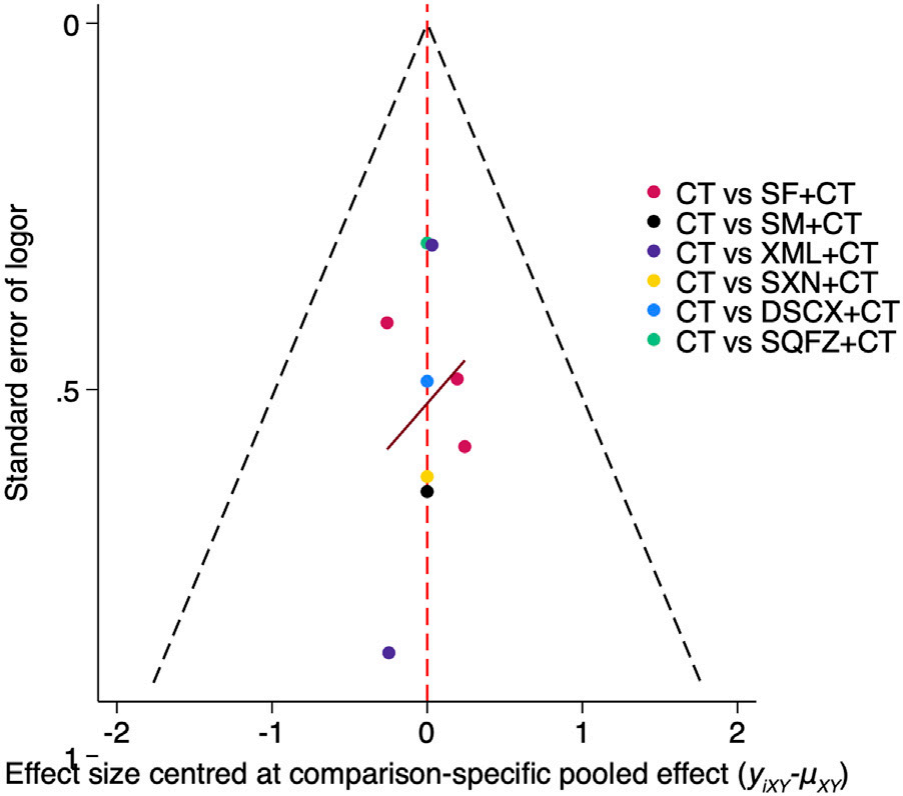
Funnel plot assessing publication bias of 28-day mortality of traditional Chinese medicine injection for treatment of SIMD.

The funnel plot for 28-day mortality (Figure 6) exhibited approximate symmetry, indicating the absence of significant publication bias. This finding was further corroborated by Egger’s linear regression test (p = 0.940 > 0.05), which confirms that there is no statistically significant publication bias associated with this outcome.

### 3.5 Secondary outcomes

#### 3.5.1 cTnI

**FIGURE 7:**
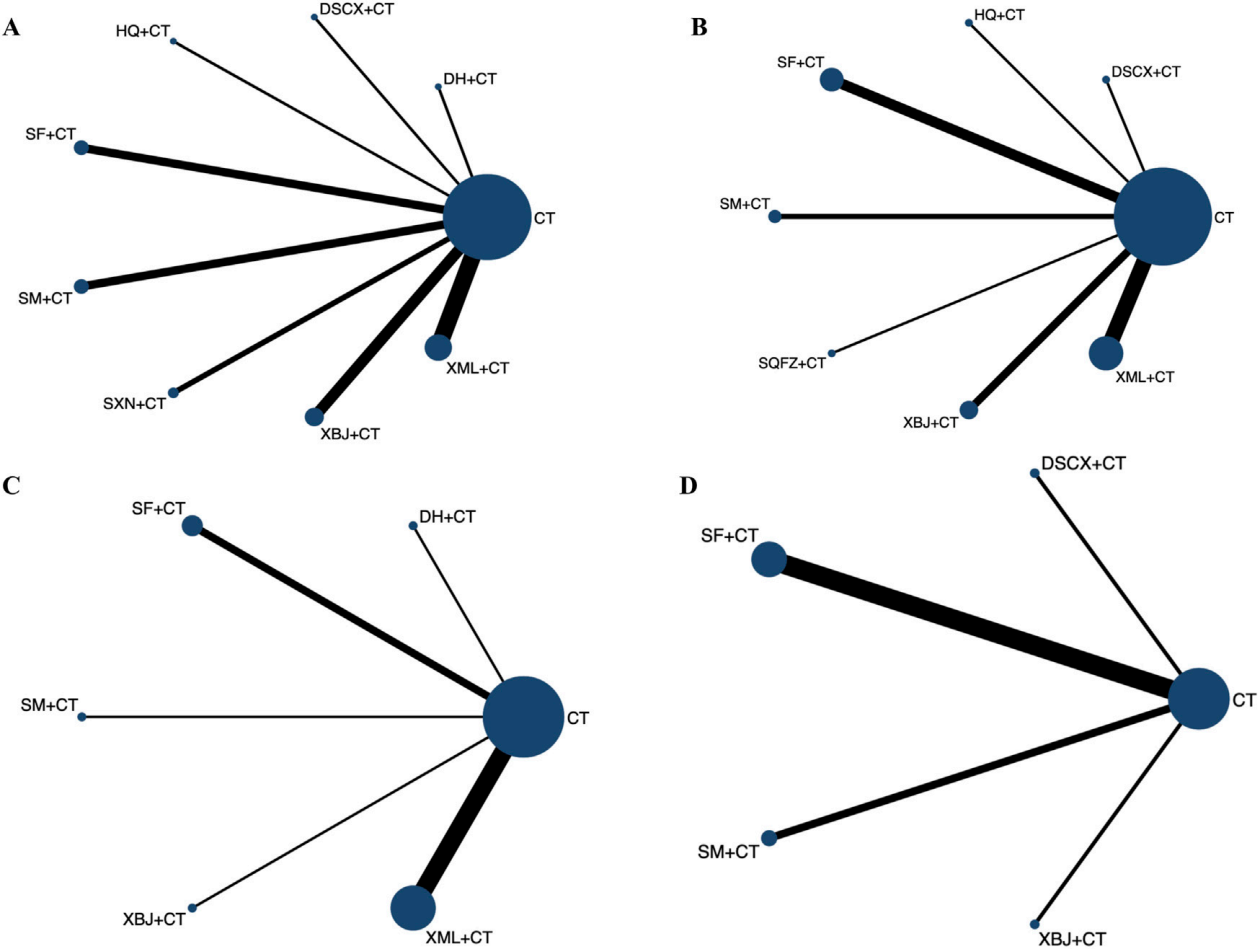
Evidence network diagram of each secondary outcome indicator of traditional Chinese medicine injection for treatment of SIMD. **(A)** cTnI; **(B)**. LVEF; **(C)**. BNP; **(D)**. NT-Pro BNP. CT, conventional therapy; SM, Shenmai injection; DSCX, Danshen Chuanxiongqin injection; XBJ, Xuebijing injection; SF, Shenfu injection; XML, Xinmailong injection; HQ, Huangqi injection; SXN, Shuxuening injection; DH, Danhong injection; SQFZ, Shenqifuzheng injection.

**FIGURE 8:**
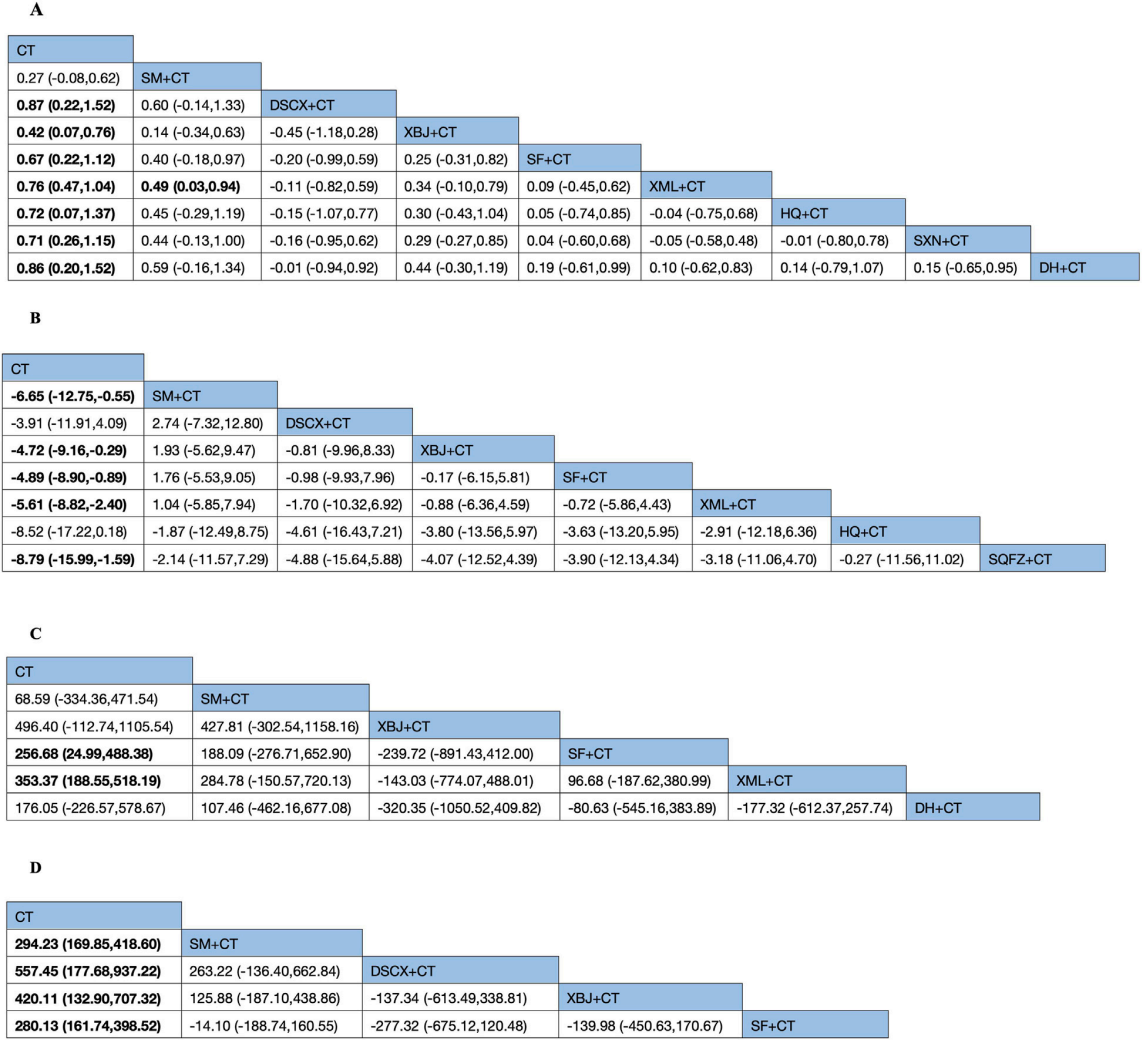
Net Meta-analysis of each secondary outcome indicator of traditional Chinese medicine injection for treatment of SIMD. **(A)** cTnI; **(B)** LVEF; **(C)** BNP; **(D)** NT-Pro BNP.

**FIGURE 9:**
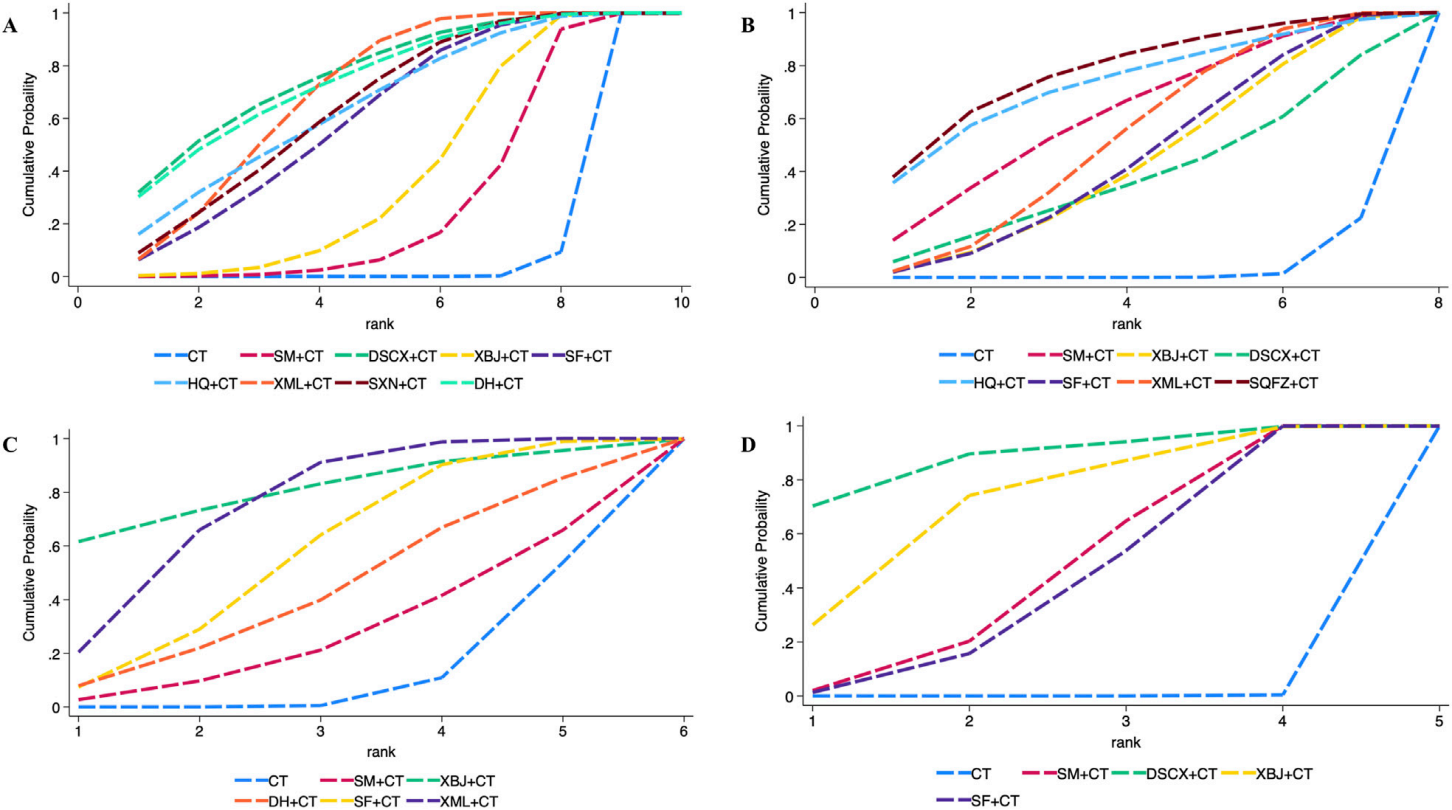
SUCRA ranking of each secondary outcome indicator of traditional Chinese medicine injection for treatment of SIMD. **(A)** cTnI; **(B)** LVEF; **(C)** BNP; **(D)** NT-Pro BNP.

cTnI was reported in 21 RCTs evaluating eight TCMIs (Figure 7A). A network meta-analysis demonstrated that DSCXQ + CT (0.87, 95% CI: 0.22 to 1.52), XBJ + CT (0.42, 95% CI: 0.07 to 0.76), SF + CT (0.67, 95% CI: 0.22 to 1.12), XML + CT (0.76, 95% CI: 0.47 to 1.04), HQ + CT (0.72, 95% CI: 0.07 to 1.37), SXN + XT (0.71, 95% CI: 0.26 to 1.15), and DH + CT (0.86, 95% CI: 0.20 to 1.52) all showed statistically significant reductions in cTnI compared to conventional therapy alone (P < 0.05). Among the indirect comparisons of different TCM injections, XML + CT was found to be more effective than SM + CT, while no significant differences were observed in all other comparisons between injections (Figure 8A). The SUCRA-based treatment hierarchy was as follows: DSCXQ + CT (73.8%) > DH + CT (73.5%) > XML + CT (67.6%) > HQ + CT (62.6%) > SXN + CT (61.4%) > SF + CT (57.3%) > XBJ + CT (32.1%) > SM + CT (20.4%) (Figure 9A).

#### 3.5.2 LVEF

LVEF was reported in 18 RCTs evaluating seven TCMIs (Figure 7B). The network meta–analysis demonstrated that the combinations of SM + CT (−6.65, 95% CI: −12.75 to −0.55), XBJ + CT (−4.72, 95% CI: −9.16 to −0.29), SF + CT (−4.89, 95% CI: −8.90 to −0.89), XML + CT (−5.61, 95% CI: −8.82 to −2.40), and SQFZ + CT (−8.79 , 95% CI: −15.99 to −1.59) all exhibited statistically significant reductions in LVEF compared to conventional therapy alone (P > 0.05). Conversely, the differences among the other TCMIs were not statistically significant (P > 0.05) (Figure 8B). The SUCRA–based treatment hierarchy was as follows: SQFZ + CT (77.4%) > HQ + CT (72.8%) > SM + CT (62.5%) > XML + CT (53.6%) > SF + CT (46.3%) > XBJ + CT (44.7%) > DSCXQ + CT (39.4%) (Figure 9B).

#### 3.5.3 BNP

BNP levels were reported in 13 RCTs evaluating five TCMIs (Figure 7C). The network meta-analysis demonstrated that the combination of SF with CT resulted in a statistically significant reduction in BNP levels (256.68, 95% CI: 24.99 to 488.38), as did the combination of XML with CT (353.37, 95% CI: 188.55 to 518.19) when compared to CT alone (P < 0.05). In contrast, the differences among the other TCM injections were not statistically significant (P > 0.05) (Figure 8C). The SUCRA-based treatment hierarchy was as follows: XBJ + CT (81.3%) > XML + CT (75.1%) > SF + CT (58.0%) > DH + CT (44.2%) > SM + CT (28.6%) (Figure 9C).

#### 3.5.4 NT-pro BNP

NT + ProBNP levels were reported in nine RCTs evaluating four TCMIs (Figure 7D). The network meta + analysis demonstrated that the combinations of SM + CT (294, 23, 95% CI: 169.85 to 418.60), DSCXQ + CT (557.45, 95% CI: 177.68 to 937.22), XBJ + CT (420.11, 95% CI: 132.90 to 707.32), XML + CT (420.11 , 95% CI: 132.90 to 707.32), and SF + CT (280.13, 95% CI: 161.74 to 398.52) all exhibited statistically significant reductions in NT + ProBNP compared to conventional therapy alone (P < 0.05). Conversely, the differences among other TCMIs were not statistically significant (P > 0.05) (Figure 8D). The SUCRA + based treatment hierarchy was as follows: DSCXQ + CT (88.5%) > 0.05 XBJ + CT (71.9%) > SM + CT (46.8%) > SF + CT (42.7%) > SM + CT (28.6%) (Figure 9D).

### 3.6 Tests of inconsistency and heterogeneity

No closed loop was formed in the study, thus consistency tests were not conducted. The heterogeneity analysis demonstrated homogeneity in 28-day mortality (*I* ^2^ = 0%) while indicating significant heterogeneity in other outcomes (*I* ^2^ > 90%). Consequently, subgroup analyses were conducted for cTnI, LVEF, BNP, and Pro-BNP by type of TCMI, yet heterogeneity remained elevated; The potential sources of heterogeneity (such as treatment duration, conventional therapy, and the dosage of different TCMIs.) could not be explicitly identified. More detailed information can be found in Supplementary Appendix Tables 10–14. We performed sensitivity analyses on all studies and discovered that the results were robust and reliable (p < 0.05). The forest plot for sensitivity analysis can be seen in Supplementary Appendix Tables 15–18.

### 3.7 Adverse events

Four studies (Feng et al., 2020; Li X. et al., 2019; Wang, 2017; He et al., 2021) reported adverse events involving two TCMIs. Due to inconsistent follow-up durations across studies, only descriptive analysis was performed as Table 2 shown.

**TABLE 2:** Adverse events.

TCM injections	Treatment group	Control group
Xuebijing injection (Feng et al., 2020)	Rash (n = 2), chest tightness (n = 1)	Rash (n = 1), Nausea and vomiting (n = 1)
Xinmailong injection (Li et al., 2019b)	0	mild pruritus (n = 1)
Xinmailong injection (Wang, 2017)	Tachycardia (n = 1)	0
Xinmailong injection (He et al., 2021)	Rash,Palpitations, Flushing, Laboratory abnormalities (n = 27)	Rash,Palpitations, Flushing, Laboratory abnormalities (n = 36)

## 4 Discussion

This study included 31 RCTs involving 2,166 participants, evaluating 9 TCMIs. The network meta-analysis results indicated that for sepsis patients with elevated myocardial enzymes, the following injections may be considered: DSCXQ, XBJ, SF, XML, DH, HQ, and SXN Injection. For elevated NT-proBNP levels, SM, SF, DSCXQ, and XBJ Injection may be considered. DSCXQ injection significantly improved levels of cTnI (MD, 0.87; 95% CI: 0.22–1.52) and NT-proBNP (MD, 557.45; 95% CI: 177.68–937.22), achieving high SUCRA rankings for both biomarkers. Nonetheless, substantial heterogeneity was observed for these outcomes, and the CINeMA framework rated the overall evidence confidence as low. Although sensitivity analyses confirmed the robustness of the results, these promising findings must consequently be interpreted with caution. For patients with elevated BNP levels, XML and SF Injection could be options. It should be noted, however, that despite its high ranking, the evidence supporting XML (MD, 353.37; 95% CI: 188.55–518.19) is characterized by substantial heterogeneity and should therefore be approached with caution. For cases primarily presenting with reduced LVEF, SM, XBJ, SF, XML, and SQFZ Injection may be considered. Despite its high ranking, the evidence for SQFZ (MD, −8.79; 95% CI: −15.99 to −1.59) warrants caution as it is derived from a single study. Regarding prognosis, SXN, SQFZ, and DSCXQ Injection might effectively reduce the 28-day mortality. SXN (RR, 3.67; 95% CI: 1.09–12.32) ranked highest for this outcome, but the very low confidence in the evidence (CINeMA) warrants cautious interpretation.

Shuxuening Injection is extracted from the botanical drug Ginkgo biloba leaves. Rong et al. (2024) found that in rat cardiomyocytes treated with Shuxuening Injection, OPA1 expression was significantly reduced while p-Drp1 expression was significantly increased. This change partially inhibits mitochondrial fission, thereby maintaining mitochondrial function and reducing the impact of energy metabolism disorders on cardiomyocytes. By regulating mitochondrial quality control, it improves the prognosis of SIMD.

Danshen Chuanxiongqin, composed of the botanical drugs Salvia miltiorrhiza and ligustrazine hydrochloride, activates the Nrf2 signaling pathway, upregulating glutathione peroxidase 4 (GPX4) expression and inhibiting ferroptosis in cardiomyocytes (Wu et al., 2024). Concurrently, it enhances the expression of antioxidant genes, such as heme oxygenase-1 (HO-1) and NAD(P)H quinone oxidoreductase-1 (NQO-1), thereby reducing oxidative stress-induced damage to myocardial mitochondria (Zhuo et al., 2021). Meanwhile, ligustrazine hydrochloride suppresses the production of inflammatory cytokines, consequently alleviating myocardial suppression (Tian et al., 2024).

Shenqi Fuzheng Injection, primarily composed of the botanical drugs Codonopsis Radix and Astragali Radix, ameliorates myocardial hemorrhage, edema, and inflammatory cell infiltration while reducing cardiomyocyte apoptosis rates (He et al., 2022). Furthermore, Li et al. (2000) showed that Codonopsis Radix maintains inotropic effects without increasing chronotropic activity (Huang and Pan, 2020).

Xinmailong injection contains an active metabolite derived from *Periplaneta americana* (American cockroach). Li et al. (2017) found that XML enhances myocardial contractility by activating T-type calcium channels and inhibiting Na+/K + -ATPase, thereby increasing intracellular calcium levels Additionally, it exhibits negative inotropic effects under specific conditions (Xue et al., 2025).

This study corroborates previous findings (Liu et al., 2023) demonstrating that adjunctive TCMIs enhance treatment efficacy for SIMD. In addition, We extended the study search to 25 June 2025 and expanded the range of included TCMIs. Furthermore, we aim to provide decision-makers with a relative efficacy ranking of all available TCMIs. The uncertainty associated with this ranking was systematically assessed using CINeMA, ensuring transparent and reliable conclusions.

Several limitations should be considered. First, the methodological quality of the included literature was suboptimal, as most studies failed to report blinding and allocation concealment, potentially introducing bias risk. Second, The CINeMA framework indicated low overall confidence in the evidence, while substantial heterogeneity was noted for several outcomes. Third, the absence of direct comparisons between certain TCMIs prevented the formation of closed loops in the network relationship diagram. Fourth, most studies lacked adequate reporting of adverse events and sufficient follow-up duration. Therefore, future high-quality, multi-center, large-sample RCTs are crucial to validate the efficacy and safety of TCMIs for SIMD.

## Data Availability

The original contributions presented in the study are included in the article/Supplementary Material, further inquiries can be directed to the corresponding authors.
