# Supplementary material for: Different traditional Chinese medicine injections combined with conventional therapy for sepsis-induced myocardial dysfunction: a systematic review and network meta-analysis
Source: Front Pharmacol. 2026 Jan 5;16:1688113. doi: 10.3389/fphar.2025.1688113 (PMC12813185; doi:10.3389/fphar.2025.1688113)
Supplement: Supplementary file 1 [file Presentation1.pdf]

Appendix

Appendix Table 1: PRISMA_2020_checklist 2
Appendix Table 2：Composition of TCMIs. 4
Appendix Table 3：More details about each TCMI. 5
Appendix Table 4：The risk of bias charts for the included studies. 24
Appendix Table 5-9:GRADE Assessment for Each Outcome 25
Table 5 : GRADE Assessment:28-day mortality 25
Table 6 : GRADE Assessment:cTnI 27
Table 7 : GRADE Assessment:LVEF 28
Table 8 : GRADE Assessment:BNP 30
Table 9: GRADE Assessment:Pro-BNP 31
Appendix Table 10-14:Analysis of Heterogeneity for Each Outcome. 32
Table 10: Heterogeneity analysis: 28-day mortality 32
Table 11: Heterogeneity analysis: cTnI 32
Table 12: Heterogeneity analysis: LVEF 33
Table 13: Heterogeneity analysis: BNP 34
Table 14: Heterogeneity analysis: NT-Pro BNP 34
Appendix Table 15-18:Sensitivity Analysis for Each Outcome. 35
Table 15: Sensitivity Analysis: cTnI 35
Table 16: Sensitivity Analysis: LVEF 35
Table 17: Sensitivity Analysis: BNP 36
Table 18: Sensitivity Analysis: NT-Pro BNP 36

Appendix Table 1: PRISMA_2020_checklist

Section and Topic	Item #	Checklist item	Location where item is reported
**TITLE**			
Title	1	Identify the report as a systematic review.	Title
**ABSTRACT**			
Abstract	2	See the PRISMA 2020 for Abstracts checklist.	Abstract
**INTRODUCTION**			
Rationale	3	Describe the rationale for the review in the context of existing knowledge.	Introduction
Objectives	4	Provide an explicit statement of the objective(s) or question(s) the review addresses.	Introduction
**METHODS**			
Eligibility criteria	5	Specify the inclusion and exclusion criteria for the review and how studies were grouped for the syntheses.	Eligibility criteria; Exclusion criteria
Information sources	6	Specify all databases, registers, websites, organisations, reference lists and other sources searched or consulted to identify studies. Specify the date when each source was last searched or consulted.	Materials and methods; Search strategy
Search strategy	7	Present the full search strategies for all databases, registers and websites, including any filters and limits used.	Search strategy
Selection process	8	Specify the methods used to decide whether a study met the inclusion criteria of the review, including how many reviewers screened each record and each report retrieved, whether they worked independently, and if applicable, details of automation tools used in the process.	Study selection and data extraction
Data collection process	9	Specify the methods used to collect data from reports, including how many reviewers collected data from each report, whether they worked independently, any processes for obtaining or confirming data from study investigators, and if applicable, details of automation tools used in the process.	Study selection and data extraction
Data items	10a	List and define all outcomes for which data were sought. Specify whether all results that were compatible with each outcome domain in each study were sought (e.g. for all measures, time points, analyses), and if not, the methods used to decide which results to collect.	Study selection and data extraction
	10b	List and define all other variables for which data were sought (e.g. participant and intervention characteristics, funding sources). Describe any assumptions made about any missing or unclear information.	Study selection and data extraction; Eligibility criteria; Exclusion criteria

Section and Topic	Item #	Checklist item	Location where item is reported
Study risk of bias assessment	11	Specify the methods used to assess risk of bias in the included studies, including details of the tool(s) used, how many reviewers assessed each study and whether they worked independently, and if applicable, details of automation tools used in the process.	risk of bias Assessment
Effect measures	12	Specify for each outcome the effect measure(s) (e.g. risk ratio, mean difference) used in the synthesis or presentation of results.	Statistical analysis
Synthesis methods	13a	Describe the processes used to decide which studies were eligible for each synthesis (e.g. tabulating the study intervention characteristics and comparing against the planned groups for each synthesis (item #5)).	Eligibility criteria; Exclusion criteria; Study selection and data extraction
	13b	Describe any methods required to prepare the data for presentation or synthesis, such as handling of missing summary statistics, or data conversions.	Study selection and data extraction
	13c	Describe any methods used to tabulate or visually display results of individual studies and syntheses.	Statistical analysis
	13d	Describe any methods used to synthesize results and provide a rationale for the choice(s). If meta-analysis was performed, describe the model(s), method(s) to identify the presence and extent of statistical heterogeneity, and software package(s) used.	Statistical analysis
	13e	Describe any methods used to explore possible causes of heterogeneity among study results (e.g. subgroup analysis, meta-regression).	Statistical analysis
	13f	Describe any sensitivity analyses conducted to assess robustness of the synthesized results.	Statistical analysis
Reporting bias assessment	14	Describe any methods used to assess risk of bias due to missing results in a synthesis (arising from reporting biases).	Statistical analysis; Risk of bias assessment
Certainty assessment	15	Describe any methods used to assess certainty (or confidence) in the body of evidence for an outcome.	Statistical analysis; Risk of bias assessment
RESULTS			
Study selection	16a	Describe the results of the search and selection process, from the number of records identified in the search to the number of studies included in the review, ideally using a flow diagram.	Study selection and study characteristics
	16b	Cite studies that might appear to meet the inclusion criteria, but which were excluded, and explain why they were excluded.	Study characteristics
Study characteristics	17	Cite each included study and present its characteristics.	Study characteristics
Risk of bias in studies	18	Present assessments of risk of bias for each included study.	Risk of bias of included studies
Results of individual studies	19	For all outcomes, present, for each study: (a) summary statistics for each group (where appropriate) and (b) an effect estimate and its precision (e.g. confidence/credible interval), ideally using structured tables or plots.	Network meta-analysis of 28-day mortality and Secondary Outcomes
Results of syntheses	20a	For each synthesis, briefly summarise the characteristics and risk of bias among contributing studies.	Confidence in evidence; Publication bias analysis; Risk of bias of included studies
	20b	Present results of all statistical syntheses conducted. If meta-analysis was done, present for each the summary estimate and its precision (e.g. confidence/credible interval) and measures of statistical heterogeneity. If comparing groups, describe the direction of the effect.	Network meta-analysis

Section and Topic	Item #	Checklist item	Location where item is reported
	20c	Present results of all investigations of possible causes of heterogeneity among study results.	Tests of inconsistency and heterogeneity
	20d	Present results of all sensitivity analyses conducted to assess the robustness of the synthesized results.	Tests of inconsistency and heterogeneity;
Reporting biases	21	Present assessments of risk of bias due to missing results (arising from reporting biases) for each synthesis assessed.	Risk of bias of included studies;Confidence in evidence ;Publication bias;
Certainty of evidence	22	Present assessments of certainty (or confidence) in the body of evidence for each outcome assessed.	Tests of inconsistency and heterogeneity; Risk of bias of included studies
DISCUSSION			
Discussion	23a	Provide a general interpretation of the results in the context of other evidence.	Discussion
	23b	Discuss any limitations of the evidence included in the review.	Discussion
	23c	Discuss any limitations of the review processes used.	Discussion
	23d	Discuss implications of the results for practice, policy, and future research.	Discussion
OTHER INFORMATION			
Registration and protocol	24a	Provide registration information for the review, including register name and registration number, or state that the review was not registered.	Materials and methods
	24b	Indicate where the review protocol can be accessed, or state that a protocol was not prepared.	Materials and methods
	24c	Describe and explain any amendments to information provided at registration or in the protocol.	Materials and methods
Support	25	Describe sources of financial or non-financial support for the review, and the role of the funders or sponsors in the review.	Declaration of competing interest
Competing interests	26	Declare any competing interests of review authors.	Credit Author Statement
Availability of data, code and other materials	27	Report which of the following are publicly available and where they can be found: template data collection forms; data extracted from included studies; data used for all analyses; analytic code; any other materials used in the review.	Supplementary Material

Appendix Table 2：Composition of TCMIs.

injection names（Chinese）	composition	source species	level of reporting in the original study
Shenmai injection (参麦注射液)	Panax ginseng C. A. Mey.<br>Ophiopogon japonicus (L. f) Ker-Gawl.	Araliaceae<br>Asparagaceae	Inadequate
Danshen Chuanxiongqin injection（丹参川芎嗪注射液）	Salvia miltiorrhiza Bunge.<br>Tetramethylpyrazine	Lamiaceae<br>Apiaceae	Inadequate
Xuebijing injection（血必净注射液）	Carthamus tinctorius L.<br>Paeonia lactiflora Pall.<br>Ligusticum chuanxiong Hort.<br>Salvia miltiorrhiza Bunge.	Asteraceae<br>Paeoniaceae<br>Apiaceae<br>Lamiaceae	Inadequate

	Angelica sinensis (Oliv.) Diels	Apiaceae	
Shenfu injection<br>（参附注射液）	Panax ginseng C. A. Mey.<br>Aconitum carmichaelii Debx.	Araliaceae<br>Ranunculaceae	Inadequate
Shuxuening injection<br>（舒血宁注射液）	Ginkgo biloba L.	Ginkgoaceae	Inadequate
Xinmailong injection<br>（心脉隆注射液）	Periplaneta americana	Blattidae	Inadequate
Huangqi injection<br>（黄芪注射液）	Astragalus mongholicus Bunge.	Fabaceae	Inadequate
Danhong injection<br>（丹红注射液）	Salvia miltiorrhiza Bunge.<br>Carthamus tinctorius L.	Lamiaceae<br>Asteraceae	Inadequate
Shenqi fuzheng injection<br>（参芪扶正注射液）	Codonopsis pilosula (Franch.) Nannf.<br>Astragalus mongholicus Bunge.	Campanulaceae<br>Fabaceae	Inadequate

Appendix Table 3：More details about each TCMI.

Implementation standards

SM	National Medical Products Administration National Drug Standard WS3-B-3428-98-2010
DSCXQ	National Medical Products Administration National Drug Standard WS-10001-（HD-1138）-2002-2017
XBJ	National Medical Products Administration National Drug Standard YBZ01242004-2010Z-2012Z
SF	National Medical Products Administration National Drug Standard WS3-B-3427-98-2013
SXN	National Medical Products Administration National Drug Standard: WS3-B-3707-98-2004-2012
XML	National Medical Products Administration National Drug Standard YBZ07062004-2009Z
HQ	National Medical Products Administration National Drug Standard WS3-B-3335-98
DH	National Medical Products Administration National Drug Standard WS-11220（ZD-1220）-2002
SQFZ	National Medical Products Administration National Drug Standard WS3-387(Z-50)-2003(Z)-2011

Note：The above information is from China Pharmaceutical Information Query Platform (recognised by the State Drug Administration of China)

Assay

**Injection**	**Identification**
SM	1. Total Saponins<br>Reference Solution Preparation: Accurately weigh an appropriate amount of ginsenoside Re reference standard and dissolve in methanol to prepare a solution containing 2 mg per mL.<br>(1) Standard Curve Preparation<br>Accurately measure 10 μL, 20 μL, 40 μL, 60 μL, 80 μL, and 100 μL of the reference solution into separate 10 mL stoppered test tubes. Evaporate the solvent completely. Precisely add 1 mL of a freshly prepared mixture of 5% vanillin-acetic acid solution and perchloric acid (2:8). Heat in a 60°C water bath for 15 minutes, then immediately cool in an ice bath. Precisely add 5 mL of glacial acetic acid and mix well. Determine the absorbance at 544 nm by ultraviolet-visible spectrophotometry (Appendix V A, Chinese Pharmacopoeia 2010 Edition, Part I). Plot the standard curve with absorbance as the y-axis and concentration as the x-axis.<br>(2) Assay<br>Accurately measure 1 mL of the test sample and apply it to a pre-treated macroporous resin column (D101, 1.5 cm × 12 cm). Elute first with 25 mL of water and discard the eluate. Then elute with 60 mL of 75% ethanol, collect the eluate, and evaporate to dryness. Dissolve the residue in ethanol and transfer to a 10 mL volumetric flask. Dilute to volume

	with ethanol and mix well to obtain the test solution. Accurately measure 1 mL of this solution into a stoppered test tube, evaporate to dryness, and proceed as described in the standard curve preparation method starting from "Precisely add 1 mL of the mixture of 5% vanillin-acetic acid solution and perchloric acid (2:8)". Determine the absorbance and calculate the concentration from the standard curve.<br>(3) Specification<br>The preparation contains 0.80–2.00 mg of total saponins, calculated as ginsenoside Re ($C_{48}H_{82}O_{18}$), per 1 mL.<br>2. Red Ginseng<br>Determine by high-performance liquid chromatography (Appendix VI D, Chinese Pharmacopoeia 2010 Edition, Part I).<br>(1) Chromatographic Conditions and System Suitability<br>Column: Waters Symmetry Shield™ RP18 (4.6 mm × 250 mm; 5.0 μm)<br>Column Temperature: 30°C<br>Mobile Phase: Acetonitrile (A) and water (B)<br>Gradient Elution Program:<br>0–30 min: A 0% → 10%, B 100% → 90%<br>30–40 min: A 10% → 23%, B 90% → 77%<br>40–50 min: A 23%, B 77%<br>50–85 min: A 23% → 60%, B 77% → 40%<br>85–95 min: A 60% → 100%, B 40% → 0%<br>Detection Wavelength: 203 nm<br>Theoretical Plates: Not less than 1,350,000, calculated for the ginsenoside Rb1 peak.<br>(2) Reference Solution Preparation<br>Accurately weigh appropriate amounts of ginsenoside Rg1, ginsenoside Re, and ginsenoside Rb1 reference standards. Dissolve in 20% acetonitrile to prepare a mixed solution containing 0.10 mg/mL ginsenoside Rg1, 0.08 mg/mL ginsenoside Re, and 0.20 mg/mL ginsenoside Rb1.<br>(3) Assay<br>Separately inject 10 μL each of the reference solution and the test sample into the liquid chromatograph. Determine the contents.<br>(4) Specification<br>The preparation contains not less than 0.10 mg of Red Ginseng, calculated as the sum of ginsenoside Rg1 ($C_{54}H_{82}O_{14}$) and ginsenoside Re ($C_{42}H_{82}O_{18}$), per 1 mL; not less than 0.10 mg, calculated as ginsenoside Rb1 ($C_{54}H_{92}O_{23}$), per 1 mL; and 0.20–0.90 mg, calculated as the sum of ginsenoside Rg1 ($C_{42}H_{72}O_{14}$), ginsenoside Re ($C_{42}H_{82}O_{18}$), and ginsenoside Rb1 ($C_{54}H_{92}O_{23}$), per 1 mL.
DSCXQ	1. Danshensu<br>Determine by High Performance Liquid Chromatography (HPLC, Appendix V D, Chinese Pharmacopoeia 2000 Edition, Part II).<br>(1) Chromatographic Conditions and System Suitability<br>Stationary Phase: Octadecylsilane-bonded silica gel<br>Mobile Phase: Methanol - 0.2% glacial acetic acid (15:85)<br>Flow Rate: 1.0 mL/min<br>Detection Wavelength: 280 nm<br>Theoretical Plates: Not less than 1,500, calculated for the danshensu peak<br>(2) Reference Solution Preparation<br>Accurately weigh an appropriate amount of sodium danshensu reference standard and dissolve in methanol. Quantitatively dilute to obtain a solution containing approximately 90 μg per mL (1 mg of sodium danshensu is

	equivalent to 0.875 mg of danshensu).<br>(3) Test Solution Preparation<br>Accurately measure 5–25 mL of the test sample into a volumetric flask. Dilute to volume with methanol and mix well. Filter through a 0.45 μm microporous membrane and use the filtrate as the test solution.<br>(4) Assay<br>Separately inject 10 μL each of the reference solution and the test solution into the chromatograph. Record the chromatograms and calculate the content by the external standard method using peak areas.<br>2. Ligustrazine Hydrochloride<br>Determine by High Performance Liquid Chromatography (HPLC, Appendix V D, Chinese Pharmacopoeia 2000 Edition, Part II).<br>(1) Chromatographic Conditions and System Suitability<br>Stationary Phase: Octadecylsilane-bonded silica gel<br>Mobile Phase: Methanol - 0.4% phosphoric acid (10:90)<br>Flow Rate: 1.0 mL/min<br>Detection Wavelength: 292 nm<br>Theoretical Plates: Not less than 2,000, calculated for the ligustrazine hydrochloride peak<br>(2) Reference Solution Preparation<br>Accurately weigh an appropriate amount of ligustrazine hydrochloride reference standard and dissolve in methanol. Quantitatively dilute to obtain a solution containing approximately 40 μg per mL.<br>(3) Test Solution Preparation<br>Accurately measure 1 mL of the test sample into a 50 mL volumetric flask. Dilute to volume with methanol and mix well. Transfer 1 mL of this solution to a 10 mL volumetric flask, dilute to volume with methanol, and mix well. Filter through a 0.45 μm microporous membrane and use the filtrate as the test solution.<br>(4) Assay<br>Separately inject 5 μL each of the reference solution and the test solution into the chromatograph. Record the chromatograms and calculate the content by the external standard method using peak areas.<br>3. Specification<br>The sterile aqueous solution contains 0.36–0.44 mg of Danshen, calculated as danshensu ($C_9H_{10}O_5$), per 1 mL.
XBJ	NA
SF	1. Reference Solution Preparation<br>Accurately weigh an appropriate amount of ginsenoside Rb1 reference standard. Dissolve in methanol to prepare a solution containing 3 mg per mL.<br>2. Standard Curve Preparation<br>Accurately pipette 20, 30, 40, 50, and 60 μL of the reference solution into separate stoppered test tubes. Evaporate the solvent on a water bath and cool. Precisely add 0.2 mL of 5% vanillin-acetic acid solution and 0.8 mL of perchloric acid to each tube. Mix well and heat in a 60°C water bath for 15 minutes. Remove and cool in an ice-water bath. Add 5 mL of glacial acetic acid and mix well. Using the corresponding reagent as a blank, determine the absorbance at 550 nm by spectrophotometry (Appendix V B). Plot the standard curve with absorbance as the ordinate and concentration as the abscissa.<br>3. Assay<br>Accurately pipette 10 mL of the test sample into a separatory funnel. Extract by shaking with chloroform (4 × 10 mL) and discard the chloroform layers. Extract the aqueous layer with water-saturated n-butanol (4 × 10 mL). Combine the n-butanol layers and wash with n-butanol-saturated water (2 × 10 mL). Discard the aqueous layers, evaporate the n-butanol layer to dryness on a water bath, dissolve the residue in methanol, and quantitatively transfer to a 5 mL

	volumetric flask. Dilute to volume with methanol and mix well to obtain the test solution. Separately, accurately measure 10 mL of a 0.2% (g/mL) polysorbate 80 solution and process it in the same manner to prepare a blank control solution. Accurately pipette 50 μL of the test solution into a stoppered test tube. Starting from "Evaporate the solvent on a water bath" in the standard curve preparation method, proceed to determine the absorbance. Calculate the content.<br>4. Specification<br>The preparation contains not less than 0.5 mg of total ginsenosides, calculated as ginsenoside Rb1 ($C_{54}H_{92}O_{23}$), per 1 mL.
SXN	1.Total Flavonol Glycosides Assay<br>(1) Content Determination: Analyzed by HPLC (Chinese Pharmacopoeia).<br>(2) Chromatographic Conditions: C18 column; mobile phase: methanol-0.4% phosphoric acid (55:45); detection wavelength: 368 nm. System suitability: Theoretical plates ≥2500 (quercetin peak); resolution ≥1.5 (quercetin vs. isorhamnetin).<br>(3) Standard Solution: Prepared by dissolving quercetin, kaempferol, and isorhamnetin reference standards (pre-dried over P2O5) in methanol to final concentrations of 0.03 mg/mL, 0.03 mg/mL, and 0.02 mg/mL, respectively (or prepared as individual stock solutions: quercetin 0.1 mg/mL, kaempferol 0.1 mg/mL, isorhamnetin 0.05 mg/mL; mixed before use).<br>(4) Test Solution: 10 mL sample hydrolyzed with 16 mL methanol and 6 mL 18% HCl under reflux (1.5 h), cooled, diluted to 50 mL with methanol, filtered (0.45 μm).<br>(5) Procedure: Injected 10 μL of solutions. Total flavonol glycosides calculated as (quercetin + kaempferol + isorhamnetin) × 2.51.<br>(6) Specification: Total flavonol glycosides must be 90.0–110.0% of the labeled claim.<br>2. Ginkgolide A Assay<br>(1) Content Determination: Analyzed by HPLC (Chinese Pharmacopoeia) with refractive index detection.<br>(2) Chromatographic Conditions: C18 column; mobile phase: methanol-water (30:70); theoretical plates ≥2500 (ginkgolide A peak).<br>(3) Standard Solution: Prepared by dissolving ginkgolide A reference standard in methanol (1 mg/mL).<br>(4) Test Solution: 25 mL sample acidified to pH 2 with dilute HCl, extracted with ethyl acetate (4 × 20 mL), washed with 5% NaCl (2 × 15 mL), back-extracted, dried, dissolved in methanol, and diluted to 2 mL.<br>(5) Procedure: Injected 10 μL of solutions.<br>(6) Specification: Ginkgolide A content ≥80% of the labeled claim.
XML	1. Composite Nucleotide Bases<br>Accurately transfer 1 mL of the test sample to a 50 mL volumetric flask, dissolve and dilute to volume with 0.05 mol/L glycine hydrochloride buffer (pH 3.0), and mix well. Accurately transfer 5 mL of this solution to a 100 mL volumetric flask, dilute to volume with the same buffer, and mix well. Determine the absorbance at 254 nm by ultraviolet-visible spectrophotometry (Appendix V A, Chinese Pharmacopoeia 2000 Edition, Part I). Calculate the content according to the following formula. The preparation contains 12.0–18.0 mg of composite nucleotide bases per 1 mL.<br>2. Combined Amino Acids<br>(1) Standard Curve Preparation: Accurately weigh 10 mg of glutamic acid reference standard, transfer to a 200 mL volumetric flask, dissolve in approximately 160 mL of warm water, cool, and dilute to volume with water. Mix well. Accurately transfer 0.2 mL, 0.4 mL, 0.6 mL, 0.8 mL, and 1.0 mL of this solution into separate stoppered test tubes. Adjust the volume in each tube to 1.0 mL with water. Add 1.0 mL of 0.2 mol/L citrate buffer (pH 5.0) and 1.0 mL of ninhydrin TS to each tube. Mix well, heat in a water bath for 15 minutes, then immediately cool and allow to stand for 5–10 minutes. Add 3.0 mL of 60% ethanol to each tube and mix well. Determine the absorbance at 570 nm by ultraviolet-visible spectrophotometry (Appendix V A, Chinese Pharmacopoeia 2005 Edition, Part I), using the

	corresponding reagent as a blank. Plot the standard curve with absorbance as the y-axis and concentration as the x-axis.<br>(2) Test Solution Preparation: Accurately transfer 1 mL of the test sample to a 50 mL volumetric flask, dilute to volume with water, and mix well. Separately and accurately transfer aliquots into an empty ampoule and a 10 mL volumetric flask. Add 1.0 mL of hydrochloric acid to the ampoule, seal, and hydrolyze at 110°C for 8 hours. After cooling, evaporate the hydrolyzate, dissolve the residue in water, transfer to a 10 mL volumetric flask, dilute to volume with water, and mix well (for total amino acid determination). Dilute the solution in the 10 mL volumetric flask to volume with water and mix well (for free amino acid determination).<br>(3) Assay: Accurately transfer 1 mL each of the two test solutions and water (as blank) into separate tubes. Follow the procedure described in the standard curve preparation starting from "Add 1.0 mL of 0.2 mol/L citrate buffer (pH 5.0)". Determine the absorbance of each solution. Calculate the combined amino acid content by subtracting the free amino acid content from the total amino acid content.<br>(4) The preparation contains 16.0–24.0 mg of combined amino acids, calculated as glutamic acid, per 1 mL.<br>3. Uracil, Hypoxanthine, and Inosine<br>Determine by high-performance liquid chromatography (Appendix VI D, Chinese Pharmacopoeia 2005 Edition, Part I).<br>(1) Chromatographic System and System Suitability: Use octadecylsilane-bonded silica gel as the stationary phase. Use 0.05 mol/L sodium acetate buffer (pH 5.0) as mobile phase A and a mixture of methanol–0.05 mol/L sodium acetate buffer (pH 5.0) (80:20) as mobile phase B. Perform gradient elution according to the following program:<br>Time (min) – Mobile Phase A (%) – Mobile Phase B (%)<br>0–5: 90 → 90, 10 → 10<br>5–15: 90 → 70, 10 → 30<br>15–18: 70, 30<br>Detection wavelength: 254 nm. Flow rate: 0.5 mL/min. The number of theoretical plates for the uracil peak is not less than 3,000.<br>(2) Reference Solution: Accurately weigh appropriate amounts of uracil, hypoxanthine, and inosine reference standards. Dissolve in mobile phase A to prepare a solution containing 30 μg/mL uracil, 40 μg/mL hypoxanthine, and 70 μg/mL inosine. Mix well.<br>(3) Test Solution: Accurately transfer 1 mL of the test sample to a 50 mL volumetric flask, dilute to volume with mobile phase A, and mix well.<br>(4) Assay: Separately inject 10 μL each of the reference solution and the test solution into the liquid chromatograph. Determine the contents.<br>(5) The preparation contains 1.0–1.6 mg of uracil ($C_4H_4N_2O_2$), 1.7–2.5 mg of hypoxanthine ($C_5H_4N_4O$), and 3.5–5.0 mg of inosine ($C_{10}H_{12}N_4O_5$) per 1 mL.
HQ	1.Content Determination: The sample (10 ml) was evaporated to dryness on a water bath. The residue was dissolved in 2 ml of 1% sodium hydroxide solution and passed through a D101 macroporous adsorption resin column (1 cm × 12 cm). After sequential elution with 50 ml of 1% sodium hydroxide (discarded), water (neutral eluent, ~50 ml, discarded), 30% ethanol (50 ml, discarded), and 70% ethanol (50 ml collected), the collected 70% ethanol eluate was evaporated to dryness. The residue was dissolved in methanol and diluted to 2 ml as the test solution. A reference solution was prepared by dissolving astragaloside reference standard in methanol (1 mg/ml). Thin-layer chromatography (TLC) was performed on silica gel G plates using the lower layer of chloroform-ethyl acetate-methanol-water (15:40:22:10, stored below 10°C) as the developing system. After derivatization with 20% sulfuric acid ethanol solution and heating at 105°C, the plates were scanned at λs=395 nm and λR=700 nm for quantitative analysis by TLC scanning.<br>2.Specification: The preparation contains not less than 0.08 mg of astragaloside IV ($C_{41}H_{68}O_{14}$) per 1 ml.
DH	1. Salviae Miltiorrhizae Radix et Rhizoma<br>Determine by High Performance Liquid Chromatography (HPLC, Appendix VI D, Chinese Pharmacopoeia 2000

	Edition, Part I).<br>(1) Chromatographic Conditions and System Suitability<br>Stationary Phase: Octadecylsilane-bonded silica gel<br>Mobile Phase: Methanol - 1% glacial acetic acid solution (13:87)<br>Detection Wavelength: 280 nm<br>Theoretical Plates: Not less than 5,000, calculated for the sodium danshensu peak<br>(2) Reference Solution Preparation<br>Accurately weigh appropriate amounts of sodium danshensu and protocatechualdehyde reference standards. Separately prepare aqueous solutions containing 50 μg of each per mL.<br>(3) Test Solution Preparation<br>Accurately measure 5 mL of the test sample into a 20 mL volumetric flask. Dilute to volume with water and mix well.<br>(4) Assay<br>Separately inject 10 μL each of the reference solutions and the test solution into the liquid chromatograph. Determine the contents.<br>(5) Specification<br>The preparation contains not less than 0.5 mg of Salviae Miltiorrhizae Radix et Rhizoma, calculated as the sum of danshensu ($C_9H_{10}O_5$) and protocatechualdehyde ($C_7H_6O_3$), per 1 mL.<br>2. Total Flavonoids<br>(1) Reference Solution Preparation<br>Accurately weigh 20 mg of rutin reference standard (dried to constant weight at 120°C) and transfer to a 100 mL volumetric flask. Add an appropriate amount of 50% methanol, shake to dissolve, and dilute to volume with the same solvent. Mix well (containing 0.2 mg of anhydrous rutin per mL).<br>(2) Standard Curve Preparation<br>Accurately measure 1.0, 2.0, 3.0, 4.0, and 5.0 mL of the reference solution into separate 10 mL volumetric flasks. Add 50% methanol to each to make 5 mL. Add 0.3 mL of 5% sodium nitrite solution to each, mix well, and let stand for 6 minutes. Add 0.3 mL of 10% aluminum nitrate solution, mix well, and let stand for 6 minutes. Add 4 mL of sodium hydroxide TS, then dilute to volume with 50% methanol and mix well. Using the corresponding solution as a blank, determine the absorbance at 500 nm by spectrophotometry (Appendix V B, Chinese Pharmacopoeia 2000 Edition, Part I). Plot the standard curve with absorbance as the ordinate and concentration as the abscissa.<br>(3) Assay<br>Accurately measure 5 mL of the test sample into a 100 mL volumetric flask. Dilute to volume with water and mix well. Accurately measure 1 mL of this solution into a 10 mL volumetric flask, dilute to volume with 50% methanol, and mix well to serve as the blank control. Separately, accurately measure another 1 mL into a 10 mL volumetric flask and, starting from "Add 50% methanol to make 5 mL" in the standard curve preparation method, proceed to determine the absorbance immediately. Read the weight of rutin equivalent in the test solution from the standard curve and calculate.<br>(4) Specification<br>The preparation contains not less than 5.0 mg of total flavonoids, calculated as rutin ($C_{27}H_{30}O_{16}$), per 1 mL.
SQFZ	1. Total Solids<br>(1) Accurately measure 20 mL of the test sample into an evaporating dish previously dried to constant weight at 105°C. Evaporate to near dryness on a water bath, then dry at 105°C for 4 hours. Weigh accurately after cooling, subtract the weight of sodium chloride, and calculate.<br>(2) The preparation contains not less than 13.0 mg of total solids per 1 mL.<br>2. Total Saponins<br>(1) Reference Solution: Accurately weigh 10 mg of astragaloside IV reference standard (dried to constant weight at

105°C) into a 100 mL volumetric flask. Dissolve and dilute to volume with methanol, and mix well (containing 0.1 mg of astragaloside IV per 1 mL).

(2) Test Solution: Accurately measure 50 mL of the test sample, concentrate to 15 mL on a water bath, and transfer to a separatory funnel. Extract with water-saturated n-butanol (4 × 20 mL). Combine the n-butanol layers and wash with n-butanol-saturated water (2 × 10 mL). Discard the aqueous layers, evaporate the n-butanol layer to dryness, dissolve the residue in methanol, transfer to a 20 mL volumetric flask, dilute to volume with methanol, and mix well. Filter, accurately measure 5 mL of the successive filtrate into a 20 mL volumetric flask, dilute to volume with methanol, and mix well.

(3) Assay: Accurately measure 1 mL each of the reference solution and the test solution into separate 25 mL Nessler tubes. Evaporate to dryness on a water bath, cool, add 0.4 mL of freshly prepared 5% vanillin-acetic acid solution and 1.6 mL of perchloric acid, mix well, and allow to stand for 5 minutes. Heat in a boiling water bath for 15 minutes, then immediately cool in an ice bath to room temperature. Add 8 mL of glacial acetic acid, mix well, and determine the absorbance at 538 nm by spectrophotometry (Appendix V B, Chinese Pharmacopoeia 2000 Edition, Part I). Calculate the content.

(4) The preparation contains not less than 0.12 mg of total saponins, calculated as astragaloside IV ($C_{41}H_{68}O_{14}$), per 1 mL.

3. Astragaloside IV

Determine by HPLC (Appendix VI D, Chinese Pharmacopoeia 2000 Edition, Part I).

(1) Chromatographic System: Use octadecylsilane-bonded silica gel as the stationary phase and a mixture of acetonitrile-water (36:64) as the mobile phase. An evaporative light-scattering detector is used. The number of theoretical plates for the astragaloside IV peak is not less than 3,000.

(2) Reference Solution: Accurately weigh astragaloside IV reference standard (dried at 105°C for 2 hours) and dissolve in methanol to obtain solutions containing 0.15 mg and 0.5 mg per 1 mL, respectively.

(3) Test Solution: Accurately measure 200 mL of the test sample, concentrate to about 20 mL on a water bath, and transfer to a separatory funnel. Adjust the total volume to 20 mL with water, and extract with water-saturated n-butanol (4 × 25 mL). Combine the n-butanol extracts, wash with ammonia TS (2 × 20 mL), and discard the ammonia layers. Evaporate the n-butanol layer to dryness, dissolve the residue in 3–5 mL of water, and pass through a D101 macroporous adsorption resin column (1.5 cm i.d. × 15 cm). Elute successively with 55 mL of water (discard), 30 mL of 30% ethanol (discard), 25 mL of 40% ethanol (discard), and 80 mL of 70% ethanol (collect). Evaporate the collected 70% ethanol eluate to dryness on a water bath. Dissolve the residue in methanol, transfer to a 5 mL volumetric flask, dilute to volume with methanol, and filter through a 0.45 μm membrane.

(4) Assay: Separately inject 20 μL each of the reference solutions and the test solution into the liquid chromatograph. Determine by the external standard two-point method using logarithmic equations.

(5) The preparation contains not less than 0.004 mg of Radix Astragali, calculated as astragaloside IV ($C_{41}H_{68}O_{14}$), per 1 mL.

4. Total Sugars

(1) Reference Solution: Accurately weigh an appropriate amount of anhydrous glucose reference standard (dried to constant weight at 105°C) and dissolve in water to obtain a solution containing 40 μg per 1 mL.

(2) Test Solution: Accurately measure 50 mL of the test sample, add 10 mL of hydrochloric acid, and hydrolyze in a boiling water bath for 3 hours. Cool, transfer to a 100 mL volumetric flask, rinse the container with water, combine the rinsings, dilute to volume with water, and mix well. Filter, discard the initial filtrate, accurately measure 2 mL of the successive filtrate into a 100 mL volumetric flask, dilute to volume with water, and mix well.

(3) Assay: Accurately measure 2 mL each of the reference solution and the test solution into separate 25 mL Nessler tubes. Add 1 mL of 2% phenol solution, mix well, and rapidly add 5.0 mL of sulfuric acid dropwise in an ice bath. Mix

	well, allow to stand for 5 minutes, heat in a boiling water bath for 10 minutes, then immediately cool to room temperature. Determine the absorbance at 488 ± 2 nm by spectrophotometry (Appendix V B, Chinese Pharmacopoeia 2000 Edition, Part I). Calculate the content.<br>The preparation contains not less than 3.5 mg of total sugars, calculated as anhydrous glucose ($C_6H_{12}O_6$), per 1 mL.<br>5. Sodium Chloride<br>(1) Accurately measure 10 mL of the test sample, add 30 mL of water and 0.5 mL of potassium chromate indicator, and titrate with 0.1 mol/L silver nitrate titrant. Each 1 mL of 0.1 mol/L silver nitrate titrant is equivalent to 5.844 mg of sodium chloride (NaCl).<br>(2) The preparation contains 98.0% to 104.0% of the labeled amount of sodium chloride (NaCl).

Note：The above information is from China Pharmaceutical Information Query Platform (recognised by the State Drug Administration of China)

Quality control tests for the pharmaceutical product

**Injection**	**Pharmacological interaction**
SM	1. pH Value: Shall be 5.0–6.5 (Appendix VII G, Chinese Pharmacopoeia 2010 Edition, Part I).<br>2. Colour of Solution: Accurately measure 1 mL of the test sample into a 25 mL Nessler cylinder with 10 mL graduation mark. Dilute to 10 mL with water and mix well. Compare with Yellow Standard Colour Solution No. 7 (Method 1, Appendix XI A, Chinese Pharmacopoeia 2010 Edition, Part I); the test solution is not more intensely coloured.<br>3. Residue on Ignition: Accurately measure 2 mL of the test sample and evaporate to dryness. Carry out the check as directed (Appendix IX J, Chinese Pharmacopoeia 2010 Edition, Part I): not more than 1.0% (g/mL); for Specifications 6 and 7: not more than 1.5% (g/mL).<br>4. Total Solids: Accurately measure 10 mL of the test sample into a previouslytared evaporating dish. Evaporate to dryness on a water bath, then dry at 105°C for 3 hours. Transfer to a desiccator, cool for 30 minutes, and weigh promptly and accurately. Calculate the content.<br>5.Total Solids Content: Contains 2.0%–3.5% (g/mL) of total solids. For Specifications 6 and 7: contains 2.5%–4.0% (g/mL) of total solids.<br>6. Related Substances: Complies with the relevant requirements, excluding tannins (Appendix IX S, Chinese Pharmacopoeia 2010 Edition, Part I).<br>7. Sterility Test: Complies with the test for sterility (Appendix XIII B, Chinese Pharmacopoeia 2010 Edition, Part I).<br>8.Pyrogens: Complies with the test for pyrogens (Appendix XIII A, Chinese Pharmacopoeia 2010 Edition, Part I), administered at a dosage of 2.5 mL per kg of rabbit body weight.<br>9. Abnormal Toxicity: Complies with the test for abnormal toxicity (Appendix XVIII B, Chinese Pharmacopoeia 2010 Edition, Part I), administered by intravenous injection.<br>10. Hemolysis and Aggregation: Complies with the test for hemolysis and aggregation (Appendix XVIII B, Chinese Pharmacopoeia 2010 Edition, Part I).<br>11. Allergic Reaction: Complies with the test for allergic reaction (Appendix XVIII B, Chinese Pharmacopoeia 2010 Edition, Part I).<br>12. Osmolality: For Specifications 6 and 7 only. Complies with the test for osmolality (Appendix IX G, Chinese Pharmacopoeia 2010 Edition, Part II): 280–320 mOsmol/kg.<br>13. Other Requirements: Complies with the relevant requirements for injections (Appendix I U, Chinese Pharmacopoeia 2010 Edition, Part I).
DSCXQ	1. pH Value: Shall be 3.0–3.5.

	2. Pyrogens: Complies with the test for pyrogens (Appendix XI D, Chinese Pharmacopoeia 2000 Edition, Part II), administered at a dosage of 0.3 mL per kg of rabbit body weight.<br>3. Other Requirements: Complies with the relevant requirements for injections (Appendix I B, Chinese Pharmacopoeia 2000 Edition, Part II).
XBJ	NA
SF	1. Limit of Aconitum Alkaloids:<br>(1) Standard Curve Preparation: Accurately weigh 5 mg of aconitine reference standard into a 50 mL volumetric flask. Dissolve and dilute to volume with 0.01 mol/L hydrochloric acid solution, and mix well. Accurately measure 0.00, 0.25, 0.50, 0.75, 1.00, 1.50, and 2.00 mL into separate separatory funnels. Sequentially add 2.00, 1.75, 1.50, 1.25, 1.00, 0.50, and 0.00 mL of 0.01 mol/L hydrochloric acid solution to each, respectively. Precisely add 10 mL of acetate buffer (prepared by adjusting 250 mL of 0.2 mol/L acetic acid solution to pH 3.1 with 0.2 mol/L sodium acetate solution), 2 mL of bromocresol green solution (prepare by dissolving 50 mg of bromocresol green in 1.6 mL of 0.05 mol/L sodium hydroxide solution, grinding to dissolve, diluting with water to 100 mL, and extracting three times with 30 mL chloroform each, discarding the chloroform layers), and 10 mL of chloroform to each. Shake for 3 minutes, allow to stand, and separate the chloroform layer. Determine the absorbance at 416 nm by spectrophotometry (Chinese Pharmacopoeia). Plot the standard curve with absorbance as the ordinate and concentration as the abscissa.<br>(2) Test Solution Preparation: Accurately measure 10 mL of the test sample into a separatory funnel. Adjust to pH 10–11 with ammonia TS and extract with chloroform (4 × 10 mL). Combine the chloroform extracts and evaporate to dryness. Dissolve the residue in 0.01 mol/L hydrochloric acid solution in portions and transfer to a 10 mL volumetric flask. Dilute to volume with the same solvent. Separately, accurately measure 10 mL of a 0.2% (g/mL) polysorbate 80 solution and process similarly to prepare a blank control solution.<br>(3) Assay: Accurately measure 2 mL each of the test solution and the blank control solution into separate separatory funnels. Starting from "Precisely add 10 mL of acetate buffer", proceed as described in the standard curve preparation method. Determine the absorbance and read the weight (μg) of aconitine in the test solution from the standard curve. Calculate the content.<br>(4) The preparation contains not more than 0.1 mg of Aconitum alkaloids, calculated as aconitine ($C_{34}H_{47}NO_{11}$), per 1 mL.<br>2. pH Value: Shall be 4.5–7.0 (Chinese Pharmacopoeia).<br>3. Abnormal Toxicity: Complies with the test for abnormal toxicity (Chinese Pharmacopoeia), administered by intravenous injection.<br>4. Pyrogens: Complies with the test for pyrogens (Chinese Pharmacopoeia), administered at a dosage of 2 mL per kg of rabbit body weight.<br>5. Hemolysis Test:Preparation of 2% Red Blood Cell Suspension: Collect rabbit heart blood in a container with glass beads. Shake for several minutes to remove fibrinogen, producing defibrinated blood. Add normal saline, mix well, and centrifuge. Discard the supernatant and wash the precipitated red blood cells with normal saline 3–4 times until the supernatant is no longer red after centrifugation. Dilute the obtained red blood cells with normal saline to prepare a 2% suspension. Use on the same day and mix well before use.<br>6. Test Procedure: Number 5 test tubes. Add 0.3 mL of the test sample and 2.2 mL of normal saline to tubes 1–3. Add 2.5 mL of normal saline to tube 4 (as the negative control) and 2.5 mL of distilled water to tube 5 (as the positive control). Place in an electric thermostatic incubator at 36.5 ± 0.5°C and observe for 3 hours. No hemolysis should occur.<br>7. Other Requirements: Complies with the relevant requirements for injections (Chinese Pharmacopoeia).
SXN	1. pH Value: Shall be 4.5–5.8 (Chinese Pharmacopoeia).<br>2. Flavone Aglycone Peak Area Ratio: Using the chromatogram from the test for Total Flavonol Glycosides, calculate the peak area ratio of quercetin to kaempferol. It shall be 1:1 ± 20%.

	3. Ginkgolic Acids: Concentrate 10 mL of the test sample to dryness. Dissolve the residue in 2 mL of methanol to obtain the test solution. Separately, reflux 1 g of Ginkgo Seed reference drug with 10 mL of ethanol for 30 minutes. Evaporate the ethanol and dissolve the residue in 2 mL of methanol to obtain the reference drug solution. Apply 5 µL of each solution on the same silica gel GF254 TLC plate. Develop using a mixture of benzene–n-hexane–ethanol (5:3:1). After development, remove the plate, air-dry, spray with vanillin-sulfuric acid TS, and heat at 105°C for approximately 5 minutes. The chromatogram of the test solution shows no spots at the positions corresponding to those in the chromatogram of the reference drug solution.<br>4. Proteins: To 1 mL of the test sample, adjust the pH to 6.5–7.0 with 1% sodium hydroxide solution. Add 1–3 drops of 1% tannic acid solution. No turbidity should be produced.<br>5. Tannins: To 1 mL of the test sample, add 5 mL of a freshly prepared solution of 1% egg albumin in normal saline. Allow to stand for 10 minutes (the solution must be freshly prepared). No turbidity or precipitate should be produced.<br>6. Pyrogens: Complies with the test for pyrogens (Chinese Pharmacopoeia), administered at a dosage of 5 mL per kg of rabbit body weight.<br>7. Residue on Ignition: Accurately measure 2 mL of the test sample and carry out the check as directed (Chinese Pharmacopoeia). The residue does not exceed 0.5 mg per 1 mL.<br>8. Heavy Metals: Using the residue obtained in the test for Residue on Ignition, carry out the check as directed (Chinese Pharmacopoeia). The heavy metals limit is not more than 10 ppm.<br>9. Other Requirements: Complies with the relevant requirements for injections (Chinese Pharmacopoeia).
XML	1. Color: Take 1 mL of the test sample, dilute with water to 10 mL, and mix well. Compare with Yellow Standard Color Solution No. 8 (Method 1, Appendix XI A, Chinese Pharmacopoeia 2005 Edition, Part I); the test solution is not more intensely colored.<br>2. pH Value: Shall be 5.4–6.4 (Appendix VII G, Chinese Pharmacopoeia 2005 Edition, Part I).<br>3. Proteins: To 1 mL of the test sample, add 1 mL of a freshly prepared 30% sulfosalicylic acid solution, mix well, and allow to stand for 5 minutes. No turbidity should be produced.<br>4. Resins: To 5 mL of the test sample, add 1 drop of hydrochloric acid and allow to stand for 30 minutes. No resinous matter should separate.<br>5. Oxalates: To 2 mL of the test sample, add 2–3 drops of 3% calcium chloride TS and allow to stand for 10 minutes. No turbidity or precipitate should be produced.<br>6. Potassium Ion: Take 2 mL of the test sample, carefully ignite to charring over a low flame, then ignite completely at 500–600°C. Dissolve the residue in 6% acetic acid and transfer to a 25 mL volumetric flask. Dilute to volume with water and mix well. Transfer 1 mL to a 10 mL Nessler cylinder. Add 12 drops of alkaline formaldehyde solution (adjust the pH of formaldehyde solution to 8.0–9.0 with 0.1 mol/L sodium hydroxide solution), 2 drops of 3% disodium edetate solution, and 0.5 mL of 3% sodium tetraphenylboron solution. Add water to make 10 mL as the test solution tube. Separately, transfer 0.8 mL of standard potassium ion solution (0.1 mg/mL) to another 10 mL Nessler cylinder and treat similarly as the reference solution tube. Compare the turbidity of the test solution tube with that of the reference solution tube by visual inspection; the test solution tube is not more turbid.<br>7. Heavy Metals: Take 2 mL of the test sample and carry out the check as directed (Appendix IX E, Method 2, Chinese Pharmacopoeia 2005 Edition, Part I). The heavy metals limit is not more than 5 ppm.<br>8. Arsenic Salt: Take 1.0 mL of the test sample, carefully char by heating (if charring is incomplete, moisten with a small amount of sulfuric acid and heat gently until completely charred), then ignite at 500–600°C until completely ashed. Allow to cool, add 5 mL of hydrochloric acid and 23 mL of water, and carry out the check as directed (Appendix IX F, Method 1, Chinese Pharmacopoeia 2005 Edition, Part I). The arsenic salt limit is not more than 2 ppm.<br>9. Pyrogens: Complies with the test for pyrogens (Appendix XIII D, Chinese Pharmacopoeia 2005 Edition, Part I), administered at a dosage of 1 mL per kg of rabbit body weight.

	10. Abnormal Toxicity: Dilute each 1 mL of the test sample with sodium chloride injection to 10 mL. Complies with the test for abnormal toxicity (Appendix XI C, Chinese Pharmacopoeia 2005 Edition, Part II), administered by intravenous injection.<br>11. Allergic Test: Dilute each 1 mL of the test sample with sodium chloride injection to 4 mL to obtain the test solution. Sensitize 6 healthy guinea pigs (weighing 250–350 g) by intraperitoneally injecting 0.5 mL of the test solution per animal every other day, for a total of 3 injections. Divide them equally into 2 groups of 3 animals each. Challenge them by intravenous injection of 1 mL of the test solution on day 14 and day 21 after the first sensitizing injection, respectively. No allergic reactions should occur within 30 minutes after the challenge injection. The test is considered non-compliant if any animal exhibits two or more of the following symptoms: piloerection, sneezing, retching, three consecutive coughs, or dyspnea; or exhibits any one of the following: convulsions, shock, or death.<br>12. Hemolysis and Aggregation Test:<br>(1) Preparation of 2% Red Blood Cell Suspension: Collect an appropriate amount of rabbit blood into an Erlenmeyer flask containing glass beads. Shake for 10 minutes to remove fibrinogen, producing defibrinated blood. Add 10 times the volume of normal saline, mix well, and centrifuge. Discard the supernatant and wash the precipitated red blood cells with normal saline 2–3 times until the supernatant is colorless. Prepare a 2% suspension of the obtained red blood cells with normal saline.<br>(2) Test Procedure: Number 5 clean test tubes (tubes 1–3 are the test sample tubes, tube 4 is the negative control tube, and tube 5 is the positive control tube). To tubes 1–3, add 2.5 mL of the 2% red blood cell suspension, 2.2 mL of normal saline, and 0.3 mL of the test solution, respectively. To tube 4, add 2.5 mL of the 2% red blood cell suspension and 2.5 mL of normal saline. To tube 5, add 2.5 mL of the 2% red blood cell suspension and 2.5 mL of distilled water. Mix each tube well and immediately place in a 37°C incubator. Observe initially every 15 minutes, then every hour after the first hour, for a total of 3 hours. No hemolysis or aggregation of red blood cells should occur within 3 hours.<br>13. Fingerprint:<br>(1) Take 1 mL of the test sample, dilute with water to 5 mL, and pass through a strong basic anion exchange resin column (Dowex 2, Cl^- type, 200 mesh, 1.5 cm i.d. × 2.5 cm length, packed by wet method, pre-treated successively with 10 mL of 0.5 mol/L sodium hydroxide solution and 10 mL of water). Wash the column with 10 mL of water and discard the eluate. Then elute with 0.25 mol/L acetic acid solution, discard the first 5 mL of eluate, and collect the next 25 mL of eluate. Mix well to obtain the test solution. Accurately weigh an appropriate amount of inosine reference standard and prepare a solution in water containing 50 µg per mL as the reference solution. Perform HPLC (Appendix VI D, Chinese Pharmacopoeia 2005 Edition, Part I) using octadecylsilane-bonded silica gel as the stationary phase (Agilent Zorbax SB, 5 µm, 4.6 × 150 mm), with methanol as mobile phase A and 0.05 mol/L sodium acetate buffer (pH 5.0) as mobile phase B. The flow rate is 0.6 mL per minute, the column temperature is 30°C, and use the following gradient program: 0–15 min, A 0%→20%, B 100%→80%. The detection wavelength is 254 nm. The number of theoretical plates for the inosine peak is not less than 5,000.<br>(2) Separately inject 5 µL each of the reference solution and the test solution into the liquid chromatograph and record the chromatogram within 25 minutes. The chromatogram of the test sample should be substantially consistent with the standard fingerprint. Import the test sample chromatogram into the Traditional Chinese Medicine Chromatographic Fingerprint Similarity Evaluation System (Version 2004A.0B) and calculate the similarity by comparing it with the standard fingerprint. The similarity should be not less than 0.90.<br>14. Other Requirements: Complies with the relevant requirements for injections (Appendix I U, Chinese Pharmacopoeia 2005 Edition, Part I).
HQ	1. pH Value: Shall be 6.0–7.5 (Chinese Pharmacopoeia).<br>2. Pyrogen Test: Complies with the test for pyrogens (Chinese Pharmacopoeia), administered at a dosage of 1.60 mL per kg of rabbit body weight.

	3. Hemolysis Test:Preparation of 2% Red Blood Cell Suspension: Collect rabbit heart blood in a container with glass beads. Shake for several minutes to remove fibrinogen, producing defibrinated blood. Add normal saline, mix well, and centrifuge. Discard the supernatant and wash the precipitated red blood cells with normal saline 3–4 times until the supernatant is no longer red after centrifugation. Dilute the obtained red blood cells with normal saline to prepare a 2% suspension. Use on the same day and mix well before use.<br>4. Test Procedure: Number 5 test tubes. Add 0.3 mL of the test sample and 2.2 mL of normal saline to tubes 1–3. Add 2.5 mL of normal saline to tube 4 (as the negative control) and 2.5 mL of distilled water to tube 5 (as the positive control). Then add 2.5 mL of the 2% red blood cell suspension to each tube. Place in an incubator at 36.5 ± 0.5°C and observe for 3 hours. No hemolysis should occur.<br>5. Other Requirements: Complies with the relevant requirements for injections (Chinese Pharmacopoeia).
DH	1. pH Value: Shall be 4.5–6.5 (Appendix VII G, Chinese Pharmacopoeia 2000 Edition, Part I).<br>2. Proteins: To 1 mL of the test sample, add 1–3 drops of tannic acid TS. No turbidity should be produced.<br>3. Residue on Ignition: Take 10 mL of the test sample and carry out the check as directed (Appendix IX J, Chinese Pharmacopoeia 2000 Edition, Part I). The residue does not exceed 1.5% (g/mL).<br>4. Hemolysis Test:<br>Preparation of 2% Red Blood Cell Suspension: Collect rabbit heart blood in a container with glass beads. Shake for 10 minutes to remove fibrinogen, producing defibrinated blood. Add normal saline, mix well, and centrifuge. Discard the supernatant and wash the precipitated red blood cells with normal saline 3–4 times until the supernatant is no longer red after centrifugation. Dilute the obtained red blood cells with normal saline to prepare a 2% suspension. Use on the same day and mix well before use.<br>Test Procedure: Number 5 test tubes. Add 0.3 mL of the test sample and 2.2 mL of normal saline to tubes 1–3. Add 2.5 mL of normal saline to tube 4 (as the negative control) and 2.5 mL of distilled water to tube 5 (as the positive control). Then add 2.5 mL of the 2% red blood cell suspension to each tube. Place in an incubator at 36.5 ± 0.5°C and observe for 3 hours. No hemolysis should occur.<br>5. Pyrogens: Complies with the test for pyrogens (Appendix XIII A, Chinese Pharmacopoeia 2000 Edition, Part I), administered at a dosage of 2 mL per kg of rabbit body weight.<br>6. Other Requirements: Complies with the relevant requirements for injections and the test for related substances in injections (Appendix I U and Appendix IX S, Chinese Pharmacopoeia 2000 Edition, Part I).
SQFZ	1. pH Value: Shall be 4.5–6.5 (Appendix VIII G, Chinese Pharmacopoeia 2000 Edition, Part I).<br>2. Proteins: To 1 mL of the test sample, add 1–3 drops of Tannic Acid Test Solution. No turbidity should be produced.<br>3. Tannins: To 1 mL of the test sample, add 1 drop of dilute acetic acid and 4–5 drops of Sodium Chloride–Gelatin Test Solution. No turbidity or precipitate should be produced.<br>4. Oxalates: To 2 mL of the test sample, add 2–3 drops of 3% Calcium Chloride Test Solution and allow to stand for 10 minutes. No turbidity or precipitate should be produced.<br>5. Potassium Ion: Complies with the test for related substances in injections (Appendix IX S, Chinese Pharmacopoeia 2000 Edition, Part I).<br>6. Resins: To 5 mL of the test sample, add 1 drop of hydrochloric acid and allow to stand for 30 minutes. No flocculent precipitate should separate.<br>7. Residue on Ignition: Accurately measure 10 mL of the test sample, evaporate to dryness, and carry out the check as directed (Appendix IX J, Chinese Pharmacopoeia 2000 Edition, Part I). The residue does not exceed 1.5% (g/mL).<br>8. Heavy Metals: Using the residue obtained in the test for Residue on Ignition, carry out the check as directed (Appendix IX E, Method 2, Chinese Pharmacopoeia 2000 Edition, Part I). The heavy metals limit is not more than 5 ppm.<br>9. Arsenic Salt: Transfer 2 mL of the test sample to a crucible, add 0.5 mL of nitric acid and 0.25 mL of perchloric acid.

	Heat gently over a low flame until fumes cease, then continue heating for a few more minutes. Allow to cool, add about 1 mL of water and 5–10 drops of hydrochloric acid, and evaporate to dryness on a water bath. Add 23 mL of water and warm to dissolve. Add 5 mL of hydrochloric acid. Separately, treat 2 mL of Standard Arsenic Solution in the same manner. Carry out the check as directed (Appendix IX F, Method 1, Chinese Pharmacopoeia 2000 Edition, Part I). The arsenic salt limit is not more than 1 ppm.<br>10. Pyrogens: Complies with the test for pyrogens (Appendix XIII A, Chinese Pharmacopoeia 2000 Edition, Part I), administered by slow injection at a dosage of 10 mL per kg of rabbit body weight.<br>11. Other Requirements: Complies with the relevant requirements for injections (Appendix I U, Chinese Pharmacopoeia 2000 Edition, Part I).

Note:The above information is from China Pharmaceutical Information Query Platform (recognised by the State Drug Administration of China)

Pharmacological effects

**Injection**	**Pharmacological interaction**
SM	NA
DSCXQ	It demonstrates antiplatelet aggregation, coronary artery dilation, blood viscosity reduction, erythrocyte flow acceleration, microcirculation improvement, along with anti-myocardial ischemia and infarction properties.
XBJ	This product reduces endotoxin-induced mortality in mice, improves coagulation dysfunction in rat DIC models (elevating platelet/fibrinogen levels, shortening TT/PT, enhancing platelet aggregation, reducing TXB2), alleviates endotoxin-induced hepatic toxicity in rats with concurrent SOD activation, antagonizes TNF-α elevation in endotoxin-challenged mice, enhances humoral immunity by increasing anti-sRBC antibody titers in sensitized mice, and activates reticuloendothelial phagocytosis (increasing clearance/phagocytic indices). Guinea pig allergy tests showed isolated cases of mild tremors.
SF	NA
SXN	NA
XML	This product promotes Ca^{2+} influx into myocardial cells, resulting in a mild and sustained increase in myocardial contractility. It induces vasodilation, reducing pulmonary arterial pressure and pulmonary capillary wedge pressure. The agent also dilates coronary arteries to enhance coronary blood flow while inhibiting oxygen free radical-mediated myocardial injury. Additionally, it exerts renal vasodilatory effects, increasing renal blood flow and promoting diuresis. The preparation improves microcirculation and helps correct neuroendocrine imbalance.
HQ	It demonstrates cardioprotective effects through positive inotropic action, enhanced myocardial contractility, increased coronary blood flow, cardiomyocyte protection, and improved cardiovascular function.
DH	NA
SQFZ	Mouse Carbon Clearance Test demonstrated that this product enhances the phagocytic function of mononuclear macrophages. When used in combination with cyclophosphamide, it exhibits a certain inhibitory effect on the growth of S180 sarcoma in mice.

Note：The above information is from China Pharmaceutical Information Query Platform (recognised by the State Drug Administration of China)

Adverse Reaction Monitoring Form for each TCMI

**Injection**	Manufacturer	**Batch number**	**Indications**	**Adverse reaction**

SM	China Shineway Pharmaceutical Co.	Z13021166	shock, coronary heart disease, viral myocarditis, chronic pulmonary heart disease, and granulocytopenia, presenting with the pattern of qi and yin deficiency .<br>It can enhance immune function in tumor patients. When used concomitantly with chemotherapeutic agents, it may exhibit certain synergistic effects and can reduce the toxic side effects induced by chemotherapy.	According to literature reports, isolated cases have been manifested by the following presentations:<br>1.Dermatological reactions: urticarial rash, facial flushing.<br>Cardiovascular effects: chest tightness, palpitations, tachycardia, angina pectoris.<br>2.Neurological/muscular manifestations: generalized weakness, paralysis, dizziness, headache, generalized tonic-clonic (epileptic) seizure.<br>3.Hypersensitivity reactions: anaphylactic shock.<br>4.Gastrointestinal/hepatic disturbances: nausea, vomiting, jaundice, gastrointestinal hemorrhage.<br>5.Other systemic involvement: acute impairment of hepatic and renal function, phlebitis.
	Zhengda Youthful Bao Pharmaceutical Co.	Z33020019		
DSCXQ	Guizhou Baiteng Pharmaceutical Co.	H52020959	This product is indicated for the management of the following ischemic vascular diseases:<br>1.Cerebrovascular diseases: cerebral insufficiency, cerebral thrombosis, cerebral embolism.<br>2.Cardiovascular diseases: coronary heart	The following adverse reactions may occur, which generally resolve after discontinuation of the medication:<br>1.Allergic reactions: Rash, pruritus, flushing, chills, laryngeal edema, dyspnea, palpitations, etc. Anaphylactic shock has been reported.<br>2.Systemic reactions: Chills, rigors, fever, fatigue, pallor, localized edema.<br>3.Skin and appendage disorders: Rash, pruritus, hyperhidrosis, local skin

			disease (manifested as chest tightness and angina pectoris), myocardial infarction, ischemic stroke.<br>3.Peripheral vascular disease: thromboangiitis obliterans.	reactions.<br>4.Psychiatric and nervous system disorders: Dizziness, headache, convulsions, localized numbness.<br>5.Cardiovascular disorders: Palpitations, chest tightness, cyanosis, phlebitis.<br>6.Gastrointestinal disorders: Nausea, vomiting, abdominal pain, abdominal distension.<br>7.Respiratory disorders: Chest tightness, dyspnea.<br>8.Administration site conditions: Pain, purpura.
XBJ	Tianjin Chasesun Pharmaceutical Co.	Z20040033	1. This product is indicated for febrile diseases attributed to the TCM pattern of toxin and stasis interbinding, with manifestations such as fever, dyspnea, palpitations, and restlessness.<br>2.Systemic inflammatory response syndrome (SIRS) induced by infection.<br>3.Impaired organ function stage of multiple organ dysfunction syndrome (MODS), as part of a combination therapy regimen.	Isolated cases of pruritus have been reported.
SF	China Resources Sanjiu (Ya'an) Pharmaceutical Co.	Z51020664<br>Z20043116	1.For collapse syndrome with sudden exhaustion of yang qi (manifested in septic, hemorrhagic, or fluid-depletion shock).<br>2.For patterns attributable to yang deficiency (or qi deficiency), presenting with symptoms such as palpitations, severe	Adverse reactions have been reported infrequently in the literature:<br>1.Allergic reactions: Pruritus, rash, allergic dermatitis, pallor, sensation of suffocation, dyspnea, laryngeal edema, palpitations, cyanosis, and decreased blood pressure; severe cases may progress to anaphylactic shock.<br>2.Systemic disorders: Chills, pyrexia, fatigue, hyperhidrosis, lower back

			palpitations with anxiety, wheezing and cough, epigastric pain, diarrhea, and bi syndrome (painful obstruction syndrome).	pain.<br>3.Nervous system disorders: Dizziness, headache, insomnia, tremor, convulsion, numbness of the lips and limbs.<br>4.Cardiovascular disorders: Facial flushing, palpitations, chest tightness, tachycardia, cardiac arrhythmia, blood pressure fluctuations.<br>5.Gastrointestinal disorders: Nausea, vomiting, abdominal distension, abdominal pain, diarrhea, hiccough, dry mouth, gastric discomfort, abnormal hepatic function.<br>6.Respiratory disorders: Cyanosis of the lips, cough, shortness of breath, tachypnea.<br>7.Renal and urinary disorders: Urinary retention, edema.<br>8.Others: Epistaxis, injection site reactions (redness, swelling, pain), phlebitis, visual disturbances.<br>According to an active safety surveillance study of this product conducted across 31 hospitals in China, the cumulative incidence of adverse reactions/events was 0.92‰, which falls into the "rare" frequency category. All reported events were assessed as "mild" in severity, with no "serious" adverse reactions/events identified.
SXN	Heilongjiang Jumbo Island Pharmaceutical Co.	Z23022004	This preparation is clinically indicated for ischemic cardiovascular and cerebrovascular diseases, including coronary artery disease, angina pectoris, cerebral embolism, and cerebrovascular spasm.	1.Allergic reactions: Flushing, skin rash, pruritus, urticaria, allergic dermatitis, angioedema, laryngeal edema, dyspnea, bronchospasm, suffocation, palpitations, cyanosis, hypotension, anaphylactic shock.<br>2. Systemic disorders: Chills, hyperpyrexia, pyrexia, generalized pain, hyperhidrosis, allergic purpura, coma.<br>3. Respiratory system disorders:

				Tachypnea, cough.<br>4. Cardiovascular and cerebrovascular disorders: Palpitations, chest tightness, tachycardia, hypertension. When combined with other antiplatelet or anticoagulant agents, case reports of intracranial hemorrhage have been documented.<br>5. Gastrointestinal disorders: Xerostomia, anorexia, nausea, vomiting, gastrointestinal discomfort, abdominal distension, abdominal pain, diarrhea, constipation, abnormal hepatic biochemical parameters (e.g., elevated transaminases). Cases of gastrointestinal hemorrhage have been reported.<br>6. Dermatological disorders: Subcutaneous petechiae and ecchymosis.<br>7. Neuropsychiatric disorders: Dizziness, headache, convulsions, tremors, insomnia.<br>8. Others: Phlebitis, intraocular hemorrhage, hematuria.
XML	YUNNAN TENGYAO PHARMACEUTICAL CO.	Z20060443	This product is indicated as an adjunctive therapy for chronic congestive heart failure resulting from chronic pulmonary heart disease. It may ameliorate symptoms such as palpitations, edema, shortness of breath, dull facial complexion, and cyanosis of the lips associated with chronic congestive heart failure presenting with the TCM pattern of dual deficiency of qi and yang, and internal blood stasis obstruction.	No information is available.

HQ	Harbin Shengtai Biopharmaceutical Co.	Z23020820	Viral myocarditis and cardiac dysfunction presenting with Heart Qi deficiency and blood stasis obstruction in the cardiovascular system. Hepatitis manifesting as Spleen deficiency with dampness retention syndrome.	1.Systemic reactions: Anaphylactoid reactions, anaphylactic shock, chills, pyrexia, facial pallor.<br>2.Respiratory system: Dyspnea, cyanosis, bronchospasm, cough.<br>3.Cardiovascular system: Palpitations, chest tightness.<br>4.Gastrointestinal system: Nausea, vomiting.<br>5.Dermatological reactions: Hyperhidrosis, skin rash, pruritus.<br>6.Neurological system: Dizziness, cephalalgia.
DH	Shandong Danhong Pharmaceutical Co.	Z20026866	This product is indicated for chest impediment (chest bi) and stroke caused by obstruction of static blood, with manifestations including chest pain, chest tightness, palpitations, facial deviation, slurred speech, limb numbness, and impaired mobility. It is used in the treatment of coronary heart disease, angina pectoris, myocardial infarction, static blood-type pulmonary heart disease, ischemic encephalopathy, and cerebral thrombosis.	1. Allergic reactions: Facial flushing, rash, pruritus, urticaria, laryngeal edema, dyspnea, sensation of suffocation, palpitations, cyanosis, decreased blood pressure, anaphylactic shock.<br>2. Systemic disorders: Chills, hyperpyrexia.<br>3. Cardiovascular disorders: Chest tightness, palpitations, elevated blood pressure.<br>4. Gastrointestinal disorders: Nausea, vomiting, abdominal pain, diarrhea; abnormal hepatic biochemical parameters have been reported.<br>5. Nervous system disorders: Dizziness, headache, convulsions, coma.<br>6. Others: Cases of purpura, hematuria, epistaxis, gingival bleeding, conjunctival hemorrhage, gastrointestinal hemorrhage, subcutaneous petechiae, and ecchymosis have been reported. One case of exfoliative dermatitis (severe drug eruption) has been reported.
SQFZ	Livzon Group Limin Pharmaceutical Factory	Z19990065	This product is indicated for fatigue, lack of strength, scanty speech, spontaneous sweating, and	1.Allergic Reactions: Rash, pruritus, dyspnea, flushing, anaphylactic shock.<br>2.Respiratory System: Chest tightness,

			dizziness due to lung-spleen qi deficiency. It may be used as an adjunctive therapy for lung cancer and gastric cancer presenting with the above manifestations.	tachypnea, cough.<br>3.Skin and Subcutaneous Tissue Disorders: Hyperhidrosis, maculopapular rash, urticaria, erythematous rash, skin redness, local skin reactions.<br>4.General Disorders and Administration Site Conditions: Chills, feeling cold, rigors, pyrexia, pain, malaise, fatigue, chest pain, edema.<br>5.Nervous System Disorders: Dizziness, headache, sensation of suffocation, convulsion, irritability, somnolence.<br>6.Gastrointestinal Disorders: Stomatitis, dry mouth, nausea, vomiting, abdominal pain, diarrhea, abdominal distension, gastric discomfort.<br>7.Cardiac Disorders: Palpitations, tachycardia.<br>8.Administration Site Reactions: Phlebitis, pain, rash, pruritus, numbness at the injection site.<br>9.A post-marketing safety re-evaluation study of this product was conducted in two phases: Phase I (20,000 cases) and Phase II (30,000 cases). The overall incidence of adverse reactions was 0.185% and 0.170%, respectively. A total of 88 adverse drug reactions were reported, including one serious adverse reaction (urticaria, flushing, pyrexia). No cases of anaphylactic shock were reported in this combined post-marketing surveillance cohort of 50,000 patients. However, the possibility of very rare anaphylactic shock during clinical use cannot be ruled out.<br>10.Patients without the pattern of qi deficiency may experience mild bleeding after administration.

Note: The above information is from China Pharmaceutical Information Query Platform (recognised by the State Drug Administration of China)

Appendix Table 4: The risk of bias charts for the included studies.

Study ID	Randomization process	Deviations from intended interventions	Missing outcome data	Measurement of the outcome	Selection of the reported result	Overall
Chen F2017	low risk	low risk	low risk	low risk	Some concerns	Some concerns
He C 2019	Some concerns	low risk	low risk	low risk	Some concerns	Some concerns
Yan Y 2017	Some concerns	low risk	low risk	Some concerns	Some concerns	Some concerns
Liu WY2018	low risk	low risk	low risk	low risk	Some concerns	Some concerns
He GQ2016	low risk	low risk	low risk	low risk	Some concerns	low risk
Wu JP 2022	low risk	low risk	low risk	low risk	Some concerns	Some concerns
Feng Y 2020	low risk	low risk	low risk	low risk	Some concerns	Some concerns
Chen DZ 2019	low risk	low risk	low risk	low risk	Some concerns	Some concerns
Han Y2020	Some concerns	low risk	low risk	Some concerns	Some concerns	Some concerns
Liang DY2010	Some concerns	low risk	low risk	low risk	Some concerns	Some concerns
Liu T2014	Some concerns	Some concerns	low risk	low risk	Some concerns	Some concerns
Lei C2014	low risk	low risk	low risk	low risk	low risk	low risk
Zhang B2025	Some concerns	Some concerns	low risk	low risk	Some concerns	Some concerns
Xu P2023	Some concerns	Some concerns	low risk	low risk	Some concerns	Some concerns
Li YY2022	Some concerns	low risk	low risk	low risk	Some concerns	Some concerns
Lin ME2014	low risk	low risk	low risk	low risk	Some concerns	Some concerns
Shen QQ2017	High risk	Some concerns	low risk	Some concerns	Some concerns	High risk
Luo YY2018	low risk	low risk	low risk	Some concerns	Some concerns	Some concerns

Hu YR2016	High risk	Some concerns	low risk	low risk	Some concerns	High risk
Li XH2019	Some concerns	Some concerns	low risk	Some concerns	Some concerns	Some concerns
Han B2017	low risk	Some concerns	low risk	low risk	Some concerns	Some concerns
Luo YY 2019	Some concerns	Some concerns	low risk	low risk	Some concerns	Some concerns
Wang YZ2017	low risk	low risk	low risk	low risk	Some concerns	Some concerns
Zhang L2018	Some concerns	Some concerns	low risk	Some concerns	Some concerns	Some concerns
Zhu J2019	low risk	low risk	low risk	low risk	Some concerns	Some concerns
He JZ2021	low risk	low risk	low risk	low risk	low risk	low risk
Huang MH2015	low risk	low risk	low risk	low risk	Some concerns	Some concerns
Xia R2019	low risk	Some concerns	low risk	Some concerns	Some concerns	Some concerns
QI HN2017	Some concerns	low risk	low risk	low risk	low risk	Some concerns
Tao KL2017	low risk	Some concerns	low risk	low risk	Some concerns	Some concerns
Wang JN2019	Some concerns	Some concerns	low risk	low risk	low risk	Some concerns

Appendix Table 5-9:GRADE Assessment for Each Outcome

Table 5 : GRADE Assessment:28-day mortality

Comparison	Number of studies	Within-study bias	Reporting bias	Indirectness	Imprecision	Heterogeneity	Incoherence	Confidence rating	Reason(s) for downgrading
A:B	21	Some concerns	Low risk	No concerns	No concerns	Major concerns	Some concerns	Very low	["28Within-study bias","Heterogeneity","Incoherence"]
A:C	11	Some concerns	Low risk	No concerns	No concerns	Major concerns	Some concerns	Very low	["Within-study bias","Heterogeneity","Incoherence"]
A:D	1	Some concerns	Low risk	No concerns	Major concerns	No concerns	Some concerns	Very low	["Within-study bias","Imprecision","Incoherence"]
A:E	2	Some concerns	Low risk	No concerns	Major concerns	No concerns	Some concerns	Very low	["Within-study bias","Imprecision","Incoherence"]
A:F	1	Some concerns	Low risk	No concerns	No concerns	Major concerns	Some concerns	Very low	["Within-study bias","Heterogeneity","Incoherence"]
A:G	3	Some concerns	Low risk	No concerns	No concerns	Major concerns	Some concerns	Very low	["Within-study bias","Heterogeneity","Incoherence"]

A:H	2	No concerns	Low risk	No concerns	Major concerns	No concerns	Some concerns	Low	["Imprecision","Incoherence"]
A:I	2	No concerns	Low risk	No concerns	Major concerns	No concerns	Some concerns	Low	["Imprecision","Incoherence"]
A:J	3	Some concerns	Low risk	No concerns	No concerns	No concerns	Some concerns	Very low	["Within-study bias","Incoherence"]
A:K	3	Some concerns	Low risk	No concerns	Major concerns	No concerns	Some concerns	Very low	["Within-study bias","Imprecision","Incoherence"]
B:C	0	Some concerns	Low risk	No concerns	Major concerns	No concerns	Some concerns	Very low	["Within-study bias","Imprecision","Incoherence"]
B:D	0	Some concerns	Low risk	No concerns	Major concerns	No concerns	Some concerns	Very low	["Within-study bias","Imprecision","Incoherence"]
B:E	0	Some concerns	Low risk	No concerns	Major concerns	No concerns	Some concerns	Very low	["Within-study bias","Imprecision","Incoherence"]
B:F	0	Some concerns	Low risk	No concerns	Major concerns	No concerns	Some concerns	Very low	["Within-study bias","Imprecision","Incoherence"]
B:G	0	Some concerns	Low risk	No concerns	Major concerns	No concerns	Some concerns	Very low	["Within-study bias","Imprecision","Incoherence"]
B:H	0	Some concerns	Low risk	No concerns	Major concerns	No concerns	Some concerns	Very low	["Within-study bias","Imprecision","Incoherence"]
B:I	0	No concerns	Low risk	No concerns	Major concerns	No concerns	Some concerns	Low	["Imprecision","Incoherence"]
B:J	0	Some concerns	Low risk	No concerns	Major concerns	No concerns	Some concerns	Very low	["Within-study bias","Imprecision","Incoherence"]
B:K	0	Some concerns	Low risk	No concerns	Major concerns	No concerns	Some concerns	Very low	["Within-study bias","Imprecision","Incoherence"]
C:D	0	Some concerns	Low risk	No concerns	Major concerns	No concerns	Some concerns	Very low	["Within-study bias","Imprecision","Incoherence"]
C:E	0	Some concerns	Low risk	No concerns	Major concerns	No concerns	Some concerns	Very low	["Within-study bias","Imprecision","Incoherence"]
C:F	0	Some concerns	Low risk	No concerns	Major concerns	No concerns	Some concerns	Very low	["Within-study bias","Imprecision","Incoherence"]
C:G	0	Some concerns	Low risk	No concerns	Major concerns	No concerns	Some concerns	Very low	["Within-study bias","Imprecision","Incoherence"]
C:H	0	Some concerns	Low risk	No concerns	Major concerns	No concerns	Some concerns	Very low	["Within-study bias","Imprecision","Incoherence"]
C:I	0	No concerns	Low risk	No concerns	Major concerns	No concerns	Some concerns	Low	["Imprecision","Incoherence"]
C:J	0	Some concerns	Low risk	No concerns	Major concerns	No concerns	Some concerns	Very low	["Within-study bias","Imprecision","Incoherence"]
C:K	0	Some concerns	Low risk	No concerns	Major concerns	No concerns	Some concerns	Very low	["Within-study bias","Imprecision","Incoherence"]
D:E	0	Some concerns	Low risk	No concerns	Major concerns	No concerns	Some concerns	Very low	["Within-study bias","Imprecision","Incoherence"]
D:F	0	Some concerns	Low risk	No concerns	Major concerns	No concerns	Some concerns	Very low	["Within-study bias","Imprecision","Incoherence"]

D:G	0	Some concerns	Low risk	No concerns	Major concerns	No concerns	Some concerns	Very low	["Within-study bias","Imprecision","Incoherence"]
D:H	0	Some concerns	Low risk	No concerns	Major concerns	No concerns	Some concerns	Very low	["Within-study bias","Imprecision","Incoherence"]

Table 6 : GRADE Assessment:cTnI

Comparison	Number of studies	Within-study bias	Reporting bias	Indirectness	Imprecision	Heterogeneity	Incoherence	Confidence rating	Reason(s) for downgrading
A:B	3	Some concerns	Low risk	No concerns	Major concerns	No concerns	Some concerns	Very low	["Within-study bias","Imprecision","Incoherence"]
A:C	1	No concerns	Low risk	No concerns	Major concerns	No concerns	Some concerns	Low	["Imprecision","Incoherence"]
A:D	4	Some concerns	Low risk	No concerns	Major concerns	No concerns	Some concerns	Low	["Within-study bias","Imprecision","Incoherence"]
A:E	3	Some concerns	Low risk	No concerns	Major concerns	No concerns	Some concerns	Very low	["Within-study bias","Imprecision","Incoherence"]
A:F	6	Some concerns	Low risk	No concerns	No concerns	Major concerns	Some concerns	Very low	["Within-study bias","Heterogeneity","Incoherence"]
A:G	1	Some concerns	Low risk	No concerns	Major concerns	No concerns	Some concerns	Very low	["Within-study bias","Imprecision","Incoherence"]
A:H	2	Some concerns	Low risk	No concerns	Major concerns	No concerns	Some concerns	Very low	["Within-study bias","Imprecision","Incoherence"]
A:I	1	Some concerns	Low risk	No concerns	Major concerns	No concerns	Some concerns	Very low	["Within-study bias","Imprecision","Incoherence"]
B:C	0	Some concerns	Low risk	No concerns	Major concerns	No concerns	Some concerns	Very low	["Within-study bias","Imprecision","Incoherence"]
B:D	0	Some concerns	Low risk	No concerns	Major concerns	No concerns	Some concerns	Very low	["Within-study bias","Imprecision","Incoherence"]
B:E	0	Some concerns	Low risk	No concerns	Major concerns	No concerns	Some concerns	Very low	["Within-study bias","Imprecision","Incoherence"]
B:F	0	Some concerns	Low risk	No concerns	Major concerns	No concerns	Some concerns	Very low	["Within-study bias","Imprecision","Incoherence"]
B:G	0	Some concerns	Low risk	No concerns	Major concerns	No concerns	Some concerns	Very low	["Within-study bias","Imprecision","Incoherence"]
B:H	0	Some concerns	Low risk	No concerns	Major concerns	No concerns	Some concerns	Very low	["Within-study bias","Imprecision","Incoherence"]
B:I	0	Some concerns	Low risk	No concerns	Major concerns	No concerns	Some concerns	Very low	["Within-study bias","Imprecision","Incoherence"]
C:D	0	Some concerns	Low risk	No concerns	Major concerns	No concerns	Some concerns	Very low	["Within-study bias","Imprecision","Incoherence"]
C:E	0	Some concerns	Low risk	No concerns	Major concerns	No concerns	Some concerns	Very low	["Within-study bias","Imprecision","Incoherence"]
C:F	0	No concerns	Low risk	No concerns	Major concerns	No concerns	Some concerns	Low	["Imprecision","Incoherence"]

C:G	0	Some concerns	Low risk	No concerns	Major concerns	No concerns	Some concerns	Very low	["Within-study bias","Imprecision","Incoherence"]
C:H	0	Some concerns	Low risk	No concerns	Major concerns	No concerns	Some concerns	Very low	["Within-study bias","Imprecision","Incoherence"]
C:I	0	Some concerns	Low risk	No concerns	Major concerns	No concerns	Some concerns	Very low	["Within-study bias","Imprecision","Incoherence"]
D:E	0	Some concerns	Low risk	No concerns	Major concerns	No concerns	Some concerns	Very low	["Within-study bias","Imprecision","Incoherence"]
D:F	0	Some concerns	Low risk	No concerns	Major concerns	No concerns	Some concerns	Very low	["Within-study bias","Imprecision","Incoherence"]
D:G	0	Some concerns	Low risk	No concerns	Major concerns	No concerns	Some concerns	Very low	["Within-study bias","Imprecision","Incoherence"]
D:H	0	Some concerns	Low risk	No concerns	Major concerns	No concerns	Some concerns	Very low	["Within-study bias","Imprecision","Incoherence"]
D:I	0	Some concerns	Low risk	No concerns	Major concerns	No concerns	Some concerns	Very low	["Within-study bias","Imprecision","Incoherence"]
E:F	0	Some concerns	Low risk	No concerns	Major concerns	No concerns	Some concerns	Very low	["Within-study bias","Imprecision","Incoherence"]
E:G	0	Some concerns	Low risk	No concerns	Major concerns	No concerns	Some concerns	Very low	["Within-study bias","Imprecision","Incoherence"]
E:H	0	Some concerns	Low risk	No concerns	Major concerns	No concerns	Some concerns	Very low	["Within-study bias","Imprecision","Incoherence"]
E:I	0	Some concerns	Low risk	No concerns	Major concerns	No concerns	Some concerns	Very low	["Within-study bias","Imprecision","Incoherence"]
F:G	0	Some concerns	Low risk	No concerns	Major concerns	No concerns	Some concerns	Very low	["Within-study bias","Imprecision","Incoherence"]
F:H	0	Some concerns	Low risk	No concerns	Major concerns	No concerns	Some concerns	Very low	["Within-study bias","Imprecision","Incoherence"]
F:I	0	Some concerns	Low risk	No concerns	Major concerns	No concerns	Some concerns	Very low	["Within-study bias","Imprecision","Incoherence"]
G:H	0	Some concerns	Low risk	No concerns	Major concerns	No concerns	Some concerns	Very low	["Within-study bias","Imprecision","Incoherence"]
G:I	0	Some concerns	Low risk	No concerns	Major concerns	No concerns	Some concerns	Very low	["Within-study bias","Imprecision","Incoherence"]
H:I	0	Some concerns	Low risk	No concerns	Major concerns	No concerns	Some concerns	Very low	["Within-study bias","Imprecision","Incoherence"]

Table 7 : GRADE Assessment:LVEF

Comparison	Number of studies	Within-study bias	Reporting bias	Indirectness	Imprecision	Heterogeneity	Incoherence	Confidence rating	Reason(s) for downgrading
A:H	2	Some concerns	Low risk	No concerns	No concerns	Major concerns	Some concerns	Very low	["Within-study bias","Heterogeneity","Incoherence"]

B:H	1	No concerns	Low risk	No concerns	Major concerns	No concerns	Some concerns	Low	["Imprecision","Incoherence"]
C:H	3	Some concerns	Low risk	No concerns	No concerns	Major concerns	Some concerns	Very low	["Within-study bias","Heterogeneity","Incoherence"]
D:H	4	Some concerns	Low risk	No concerns	No concerns	Major concerns	Some concerns	Very low	["Within-study bias","Heterogeneity","Incoherence"]
E:H	6	Some concerns	Low risk	No concerns	No concerns	Major concerns	Some concerns	Very low	["Within-study bias","Heterogeneity","Incoherence"]
F:H	1	Some concerns	Low risk	No concerns	No concerns	Major concerns	Some concerns	Very low	["Within-study bias","Heterogeneity","Incoherence"]
G:H	1	Some concerns	Low risk	No concerns	No concerns	Major concerns	Some concerns	Very low	["Within-study bias","Heterogeneity","Incoherence"]
A:B	0	Some concerns	Low risk	No concerns	Major concerns	No concerns	Some concerns	Very low	["Within-study bias","Imprecision","Incoherence"]
A:C	0	Some concerns	Low risk	No concerns	Major concerns	No concerns	Some concerns	Very low	["Within-study bias","Imprecision","Incoherence"]
A:D	0	Some concerns	Low risk	No concerns	Major concerns	No concerns	Some concerns	Very low	["Within-study bias","Imprecision","Incoherence"]
A:E	0	Some concerns	Low risk	No concerns	Major concerns	No concerns	Some concerns	Very low	["Within-study bias","Imprecision","Incoherence"]
A:F	0	Some concerns	Low risk	No concerns	Major concerns	No concerns	Some concerns	Very low	["Within-study bias","Imprecision","Incoherence"]
A:G	0	Some concerns	Low risk	No concerns	Major concerns	No concerns	Some concerns	Very low	["Within-study bias","Imprecision","Incoherence"]
B:C	0	No concerns	Low risk	No concerns	Major concerns	No concerns	Some concerns	Low	["Imprecision","Incoherence"]
B:D	0	No concerns	Low risk	No concerns	Major concerns	No concerns	Some concerns	Low	["Imprecision","Incoherence"]
B:E	0	No concerns	Low risk	No concerns	Major concerns	No concerns	Some concerns	Low	["Imprecision","Incoherence"]
B:F	0	Some concerns	Low risk	No concerns	Major concerns	No concerns	Some concerns	Very low	["Within-study bias","Imprecision","Incoherence"]
B:G	0	Some concerns	Low risk	No concerns	Major concerns	No concerns	Some concerns	Very low	["Within-study bias","Imprecision","Incoherence"]
C:D	0	Some concerns	Low risk	No concerns	Major concerns	No concerns	Some concerns	Very low	["Within-study bias","Imprecision","Incoherence"]
C:E	0	Some concerns	Low risk	No concerns	Major concerns	No concerns	Some concerns	Very low	["Within-study bias","Imprecision","Incoherence"]
C:F	0	Some concerns	Low risk	No concerns	Major concerns	No concerns	Some concerns	Very low	["Within-study bias","Imprecision","Incoherence"]
C:G	0	Some concerns	Low risk	No concerns	Major concerns	No concerns	Some concerns	Very low	["Within-study bias","Imprecision","Incoherence"]
D:E	0	Some concerns	Low risk	No concerns	Major concerns	No concerns	Some concerns	Very low	["Within-study bias","Imprecision","Incoherence"]

D:F	0	Some concerns	Low risk	No concerns	Major concerns	No concerns	Some concerns	Very low	["Within-study bias","Imprecision","Incoherence"]
D:G	0	Some concerns	Low risk	No concerns	Major concerns	No concerns	Some concerns	Very low	["Within-study bias","Imprecision","Incoherence"]
E:F	0	Some concerns	Low risk	No concerns	Major concerns	No concerns	Some concerns	Very low	["Within-study bias","Imprecision","Incoherence"]
E:G	0	Some concerns	Low risk	No concerns	Major concerns	No concerns	Some concerns	Very low	["Within-study bias","Imprecision","Incoherence"]
F:G	0	Some concerns	Low risk	No concerns	Major concerns	No concerns	Some concerns	Very low	["Within-study bias","Imprecision","Incoherence"]
A:H	2	Some concerns	Low risk	No concerns	No concerns	Major concerns	Some concerns	Very low	["Within-study bias","Heterogeneity","Incoherence"]
B:H	1	No concerns	Low risk	No concerns	Major concerns	No concerns	Some concerns	Low	["Imprecision","Incoherence"]
C:H	3	Some concerns	Low risk	No concerns	No concerns	Major concerns	Some concerns	Very low	["Within-study bias","Heterogeneity","Incoherence"]
D:H	4	Some concerns	Low risk	No concerns	No concerns	Major concerns	Some concerns	Very low	["Within-study bias","Heterogeneity","Incoherence"]
E:H	6	Some concerns	Low risk	No concerns	No concerns	Major concerns	Some concerns	Very low	["Within-study bias","Heterogeneity","Incoherence"]
F:H	1	Some concerns	Low risk	No concerns	No concerns	Major concerns	Some concerns	Very low	["Within-study bias","Heterogeneity","Incoherence"]
G:H	1	Some concerns	Low risk	No concerns	No concerns	Major concerns	Some concerns	Very low	["Within-study bias","Heterogeneity","Incoherence"]
A:B	0	Some concerns	Low risk	No concerns	Major concerns	No concerns	Some concerns	Very low	["Within-study bias","Imprecision","Incoherence"]

Table 8 : GRADE Assessment:BNP

Comparison	Number of studies	Within-study bias	Reporting bias	Indirectness	Imprecision	Heterogeneity	Incoherence	Confidence rating	Reason(s) for downgrading
A:F	1	Some concerns	Low risk	No concerns	Major concerns	No concerns	Some concerns	Very low	["Within-study bias","Imprecision","Incoherence"]
B:F	1	Some concerns	Low risk	No concerns	Major concerns	No concerns	Some concerns	Very low	["Within-study bias","Imprecision","Incoherence"]
C:F	3	Major concerns	Low risk	No concerns	Major concerns	No concerns	Some concerns	Very low	["Within-study bias","Imprecision","Incoherence"]
D:F	7	Some concerns	Low risk	No concerns	No concerns	Major concerns	Some concerns	Very low	["Within-study bias","Heterogeneity","Incoherence"]
E:F	1	Some concerns	Low risk	No concerns	Major concerns	No concerns	Some concerns	Very low	["Within-study bias","Imprecision","Incoherence"]
A:B	0	Some concerns	Low risk	No concerns	Major concerns	No concerns	Some concerns	Very low	["Within-study bias","Imprecision","Incoherence"]

A:C	0	Some concerns	Low risk	No concerns	Major concerns	No concerns	Some concerns	Very low	["Within-study bias","Imprecision","Incoherence"]
A:D	0	Some concerns	Low risk	No concerns	Major concerns	No concerns	Some concerns	Very low	["Within-study bias","Imprecision","Incoherence"]
A:E	0	Some concerns	Low risk	No concerns	Major concerns	No concerns	Some concerns	Very low	["Within-study bias","Imprecision","Incoherence"]
B:C	0	Some concerns	Low risk	No concerns	Major concerns	No concerns	Some concerns	Very low	["Within-study bias","Imprecision","Incoherence"]
B:D	0	Some concerns	Low risk	No concerns	Major concerns	No concerns	Some concerns	Very low	["Within-study bias","Imprecision","Incoherence"]
B:E	0	Some concerns	Low risk	No concerns	Major concerns	No concerns	Some concerns	Very low	["Within-study bias","Imprecision","Incoherence"]
C:D	0	Some concerns	Low risk	No concerns	Major concerns	No concerns	Some concerns	Very low	["Within-study bias","Imprecision","Incoherence"]
C:E	0	Some concerns	Low risk	No concerns	Major concerns	No concerns	Some concerns	Very low	["Within-study bias","Imprecision","Incoherence"]
D:E	0	Some concerns	Low risk	No concerns	Major concerns	No concerns	Some concerns	Very low	["Within-study bias","Imprecision","Incoherence"]

Table 9: GRADE Assessment:Pro-BNP

Comparison	Number of studies	Within-study bias	Reporting bias	Indirectness	Imprecision	Heterogeneity	Incoherence	Confidence rating	Reason(s) for downgrading
A:E	2	Some concerns	Low risk	No concerns	No concerns	Major concerns	Some concerns	Very low	["Within-study bias","Heterogeneity","Incoherence"]
B:E	1	No concerns	Low risk	No concerns	No concerns	Major concerns	Some concerns	Low	["Heterogeneity","Incoherence"]
C:E	1	Some concerns	Low risk	No concerns	No concerns	Major concerns	Some concerns	Very low	["Within-study bias","Heterogeneity","Incoherence"]
D:E	5	Some concerns	Low risk	No concerns	No concerns	Major concerns	Some concerns	Very low	["Within-study bias","Heterogeneity","Incoherence"]
A:B	0	Some concerns	Low risk	No concerns	Major concerns	No concerns	Some concerns	Very low	["Within-study bias","Imprecision","Incoherence"]
A:C	0	Some concerns	Low risk	No concerns	Major concerns	No concerns	Some concerns	Very low	["Within-study bias","Imprecision","Incoherence"]
A:D	0	Some concerns	Low risk	No concerns	Major concerns	No concerns	Some concerns	Very low	["Within-study bias","Imprecision","Incoherence"]
B:C	0	Some concerns	Low risk	No concerns	Major concerns	No concerns	Some concerns	Very low	["Within-study bias","Imprecision","Incoherence"]
B:D	0	Some concerns	Low risk	No concerns	Major concerns	No concerns	Some concerns	Very low	["Within-study bias","Imprecision","Incoherence"]
C:D	0	Some concerns	Low risk	No concerns	Major concerns	No concerns	Some concerns	Very low	["Within-study bias","Imprecision","Incoherence"]

Appendix Table 10-14:Analysis of Heterogeneity for Each Outcome.

Table 10: Heterogeneity analysis: 28-day mortality

Study	RR (95% CI)	Weight (%)
He C2019	0.60 (0.17, 2.10)	4.93
Subtotal (I-squared = .%, p = .)	0.60 (0.17, 2.10)	4.93
He GQ2016	0.32 (0.12, 0.83)	14.05
Subtotal (I-squared = .%, p = .)	0.32 (0.12, 0.83)	14.05
Liu T2014	1.05 (0.34, 3.26)	4.93
Lei C2024	1.00 (0.39, 2.59)	6.90
Shen QQ2017	0.64 (0.29, 1.42)	10.85
Subtotal (I-squared = 0.0%, p = 0.691)	0.84 (0.49, 1.43)	22.68
Zhu J2019	0.61 (0.11, 3.26)	3.10
He JZ2021	0.80 (0.44, 1.45)	19.73
Subtotal (I-squared = 0.0%, p = 0.760)	0.77 (0.44, 1.35)	22.82
Xia R2019	0.27 (0.08, 0.92)	10.85
Subtotal (I-squared = .%, p = .)	0.27 (0.08, 0.92)	10.85
Wang JN2019	0.44 (0.24, 0.79)	24.66
Subtotal (I-squared = .%, p = .)	0.44 (0.24, 0.79)	24.66
Overall	0.58 (0.44, 0.77)	100.00
Heterogeneity chi-squared = 7.37 (d.f. = 8) p = 0.497		
I-squared (variation in RR attributable to heterogeneity) = 0.0%		
Test of RR=1 : z= 3.76 p = 0.000		

Table 11: Heterogeneity analysis: cTnI

Study	SMD (95% CI)	Weight (%)
Yan Y 2014	-4.43 (-5.09, -3.77)	4.87
Liu WY 2018	-3.42 (-4.02, -2.82)	4.95
Chen F 2017	0.85 (0.03, 1.68)	4.66
Subtotal (I-squared = 98.1%, p = 0.000)	-2.34 (-5.20, 0.51)	14.48
He GQ 2016	-1.37 (-1.78, -0.97)	5.14
Subtotal (I-squared = .%, p = .)	-1.37 (-1.78, -0.97)	5.14
Feng Y 2020	-1.26 (-1.82, -0.71)	5.00
Chen DZ 2019	-0.29 (-0.80, 0.22)	5.05
Liang DY 2010	-19.38 (-24.80, -13.95)	0.74
Han Y 2020	-0.60 (-1.11, -0.08)	5.04
Subtotal (I-squared = 94.2%, p = 0.000)	-1.85 (-3.34, -0.37)	15.82
Xu P 2023	-0.27 (-0.90, 0.36)	4.91
Luo YY 2018	-1.42 (-1.99, -0.85)	4.98
Hu YR 2016	-1.96 (-2.56, -1.36)	4.95
Subtotal (I-squared = 86.7%, p = 0.001)	-1.22 (-2.17, -0.27)	14.84
Li XH 2019	-1.33 (-1.89, -0.77)	4.99
Han B 2017	-1.62 (-2.19, -1.05)	4.98

Luo YY 2019	-1.42 (-1.99, -0.85)	4.98
Wang YZ 2017	-3.03 (-4.00, -2.06)	4.44
Zhang L 2018	-2.07 (-2.76, -1.39)	4.84
He JZ 2021	0.03 (-0.26, 0.31)	5.23
Subtotal (I-squared = 93.9%, p = 0.000)	-1.53 (-2.43, -0.63)	29.47
Huang MH 2015	-1.41 (-1.96, -0.86)	5.00
Subtotal (I-squared = .%, p = .)	-1.41 (-1.96, -0.86)	5.00
Xia R 2019	-1.66 (-2.13, -1.20)	5.09
Qi HN 2017	-1.40 (-1.87, -0.92)	5.08
Subtotal (I-squared = 0.0%, p = 0.426)	-1.53 (-1.86, -1.20)	10.17
Tao KL 2017	-1.36 (-1.85, -0.88)	5.07
Subtotal (I-squared = .%, p = .)	-1.36 (-1.85, -0.88)	5.07
D+L pooled SMD	-1.60 (-2.10, -1.09)	100.00
Heterogeneity chi-squared = 345.09 (d.f. = 20) p = 0.000		
I-squared (variation in SMD attributable to heterogeneity) = 94.2%		
Estimate of between-study variance Tau-squared = 1.2384		
Test of SMD=0 : z= 6.22 p = 0.000		
NOTE: Weights are from random effects analysis		

Table 12: Heterogeneity analysis: LVEF

Study	SMD (95% CI)	Weight (%)
Liu WY2018	0.80 (0.40, 1.19)	5.91
Chen F2017	0.84 (0.02, 1.66)	5.39
Subtotal (I-squared = 0.0%, p = 0.931)	0.81 (0.45, 1.16)	11.29
He GQ2016	0.43 (0.04, 0.81)	5.92
Subtotal (I-squared = .%, p = .)	0.43 (0.04, 0.81)	5.92
Wu JP 2022	0.11 (-0.38, 0.60)	5.81
Feng Y 2020	0.79 (0.27, 1.32)	5.78
Chen DZ 2019	1.95 (1.33,2.56)	5.67
Subtotal (I-squared = 90.3%, p = 0.000)	0.94 (-0.07, 1.95)	17.26
Liu T2014	0.55 (0.06, 1.03)	5.82
Lei C2014	0.35 (-0.09, 0.79)	5.87
Zhang B2025	0.55 (0.10,0.99)	5.87
Luo YY2018	1.39 (0.83, 1.96)	5.73
Subtotal (I-squared = 65.9%, p = 0.032)	0.68 (0.27, 1.09)	23.29
Li XH2019	6.44 (5.16, 7.71)	4.63
Han B2017	0.61 (0.10, 1.11)	5.81
Luo YY 2019	1.53 (0.96, 2.11)	5.72
Wang YZ2017	0.16 (-0.49, 0.82)	5.62
Zhu J2019	3.87 (2.84,4.89)	5.05
He JZ2021	-0.04 (-0.33,0.24)	6.00
Subtotal (I-squared = 96.7%, p = 0.000)	1.98 (0.68, 3.28)	32.82
Huang MH2015	0.85 (0.34, 1.37)	5.79

Subtotal (I-squared =.%, p =.)	0.85 (0.34, 1.37)	5.79
Wang JN2019	12.98 (11.10, 14.85)	3.62
Subtotal (I-squared = .%, p = .)	12.98 (11.10,14.85)	3.62
D+L pooled SMD	1.59 (1.03,2.15)	100.00
Heterogeneity chi-squared = 348.22 (d.f. = 17) p = 0.000		
I-squared (variation in SMD attributable to heterogeneity) = 95.1%		
Estimate of between-study variance Tau-squared = 1.3449		
Test of SMD=0 : z= 5.54 p = 0.000		
NOTE: Weights are from random effects analysis		

Table 13: Heterogeneity analysis: BNP

Study	SMD (95% CI)	Weight (%)
Chen F2017	-0.90 (-1.72, -0.07)	7.95
Subtotal (I-squared = .%, p = .)	-0.90 (-1.72, -0.07)	7.95
Han Y2020	-0.55 (-1.06, -0.03)	8.30
Subtotal (I-squared = .%, p = .)	-0.55 (-1.06, -0.03)	8.30
Shen QQ2017	-3.44 (-4.24, -2.63)	7.97
Luo YY2018	-2.54 (-3.23, -1.86)	8.12
Hu YR2016	-3.10 (-3.83, -2.37)	8.06
Subtotal (I-squared = 31.5%, p = 0.232)	-2.99 (-3.51, -2.48)	24.16
Li XH2019	6.14 (-7.36, -4.91)	7.33
Han B2017	1.76 (-2.34, -1.18)	8.23
Luo YY 2019	2.55 (-3.24, -1.87)	8.12
Wang YZ2017	2.06 (-2.88, -1.24)	7.96
Zhang L2018	2.38 (-3.11, -1.66)	8.07
Zhu J2019	18.33 (-22.34, -14.32)	3.10
He JZ2021	0.13 (-0.42, 0.15)	8.46
Subtotal (I-squared = 97.3%, p = 0.000)	3.71 (-5.29, -2.13)	51.28
Tao KL2017	1.41 (-1.90, -0.92)	8.32
Subtotal (I-squared = .%, p = .)	1.41 (-1.90, -0.92)	8.32
D+L pooled SMD	-2.70 (-3.59, -1.82)	100.00
Heterogeneity chi-squared = 292.46 (d.f. = 12) p = 0.000		
I-squared (variation in SMD attributable to heterogeneity) = 95.9%		
Estimate of between-study variance Tau-squared = 2.3856		
Test of SMD=0 : z= 5.99 p = 0.000		
NOTE: Weights are from random effects analysis		

Table 14: Heterogeneity analysis: NT-Pro BNP

Study	SMD (95% CI)	Weight (%)
Yan Y 2017	-3.37 (-3.93, -2.82)	10.94
Liu WY2018	-0.66 (-1.05, -0.27)	11.60
Subtotal (I-squared = 98.4%, p = 0.000)	-2.01 (-4.67, 0.65)	22.54

He GQ2016	-0.59 (-0.96, -0.21)	11.66
Subtotal (I-squared = .%, p = .)	-0.59 (-0.96, -0.21)	11.66
Chen DZ 2019	-0.87 (-1.40, -0.34)	11.05
Subtotal (I-squared = .%, p = .)	-0.87 (-1.40, -0.34)	11.05
Liu T2014	-0.89 (-1.39, -0.39)	11.18
Zhang B2025	-0.54 (-0.98, -0.10)	11.41
Xu P2023	-0.53 (-1.17, 0.11)	10.54
Li YY2022	-0.78 (-1.37, -0.18)	10.75
Lin ME2014	-1.42 (-1.99, -0.85)	10.87
Subtotal (I-squared = 40.6%, p = 0.151)	-0.82 (-1.14, -0.51)	54.76
D+L pooled SMD	-1.07 (-1.61, -0.52)	100.00
Heterogeneity chi-squared = 87.60 (d.f. = 8) p = 0.000		
I-squared (variation in SMD attributable to heterogeneity) = 90.9%		
Estimate of between-study variance Tau-squared = 0.6245		
Test of SMD=0 : z= 3.84 p = 0.000		
NOTE: Weights are from random effects analysis		

Appendix Table 15-18:Sensitivity Analysis for Each Outcome.

Table 15: Sensitivity Analysis: cTnI

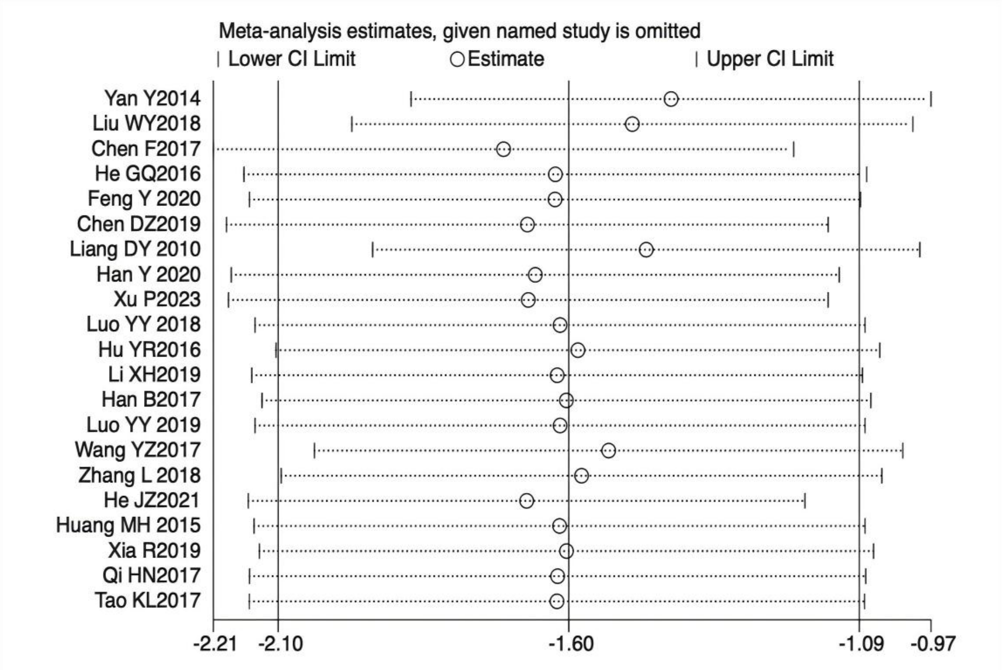

Table 16: Sensitivity Analysis: LVEF

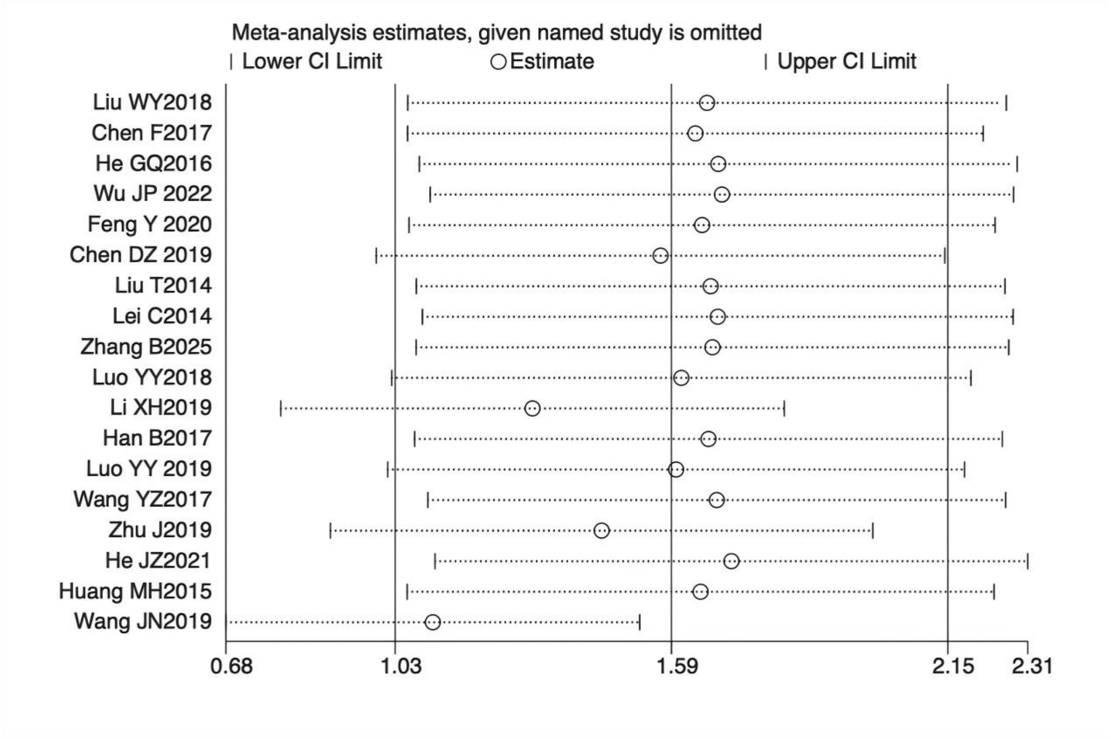

Table 17: Sensitivity Analysis: BNP

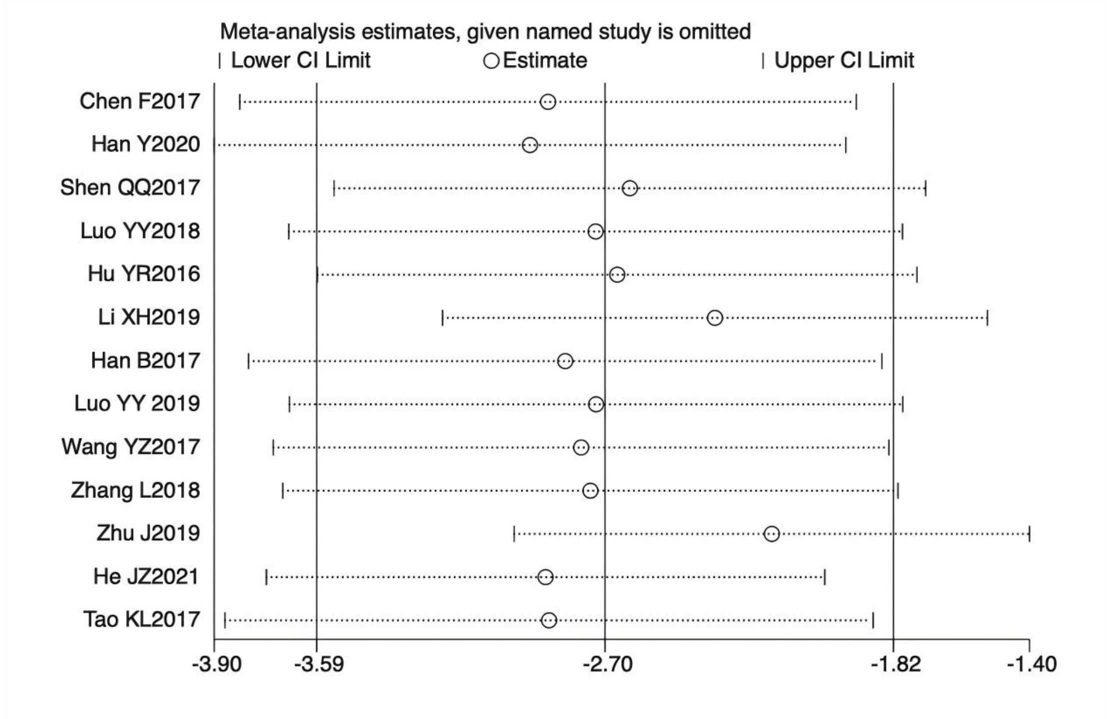

Table 18: Sensitivity Analysis: NT-Pro BNP

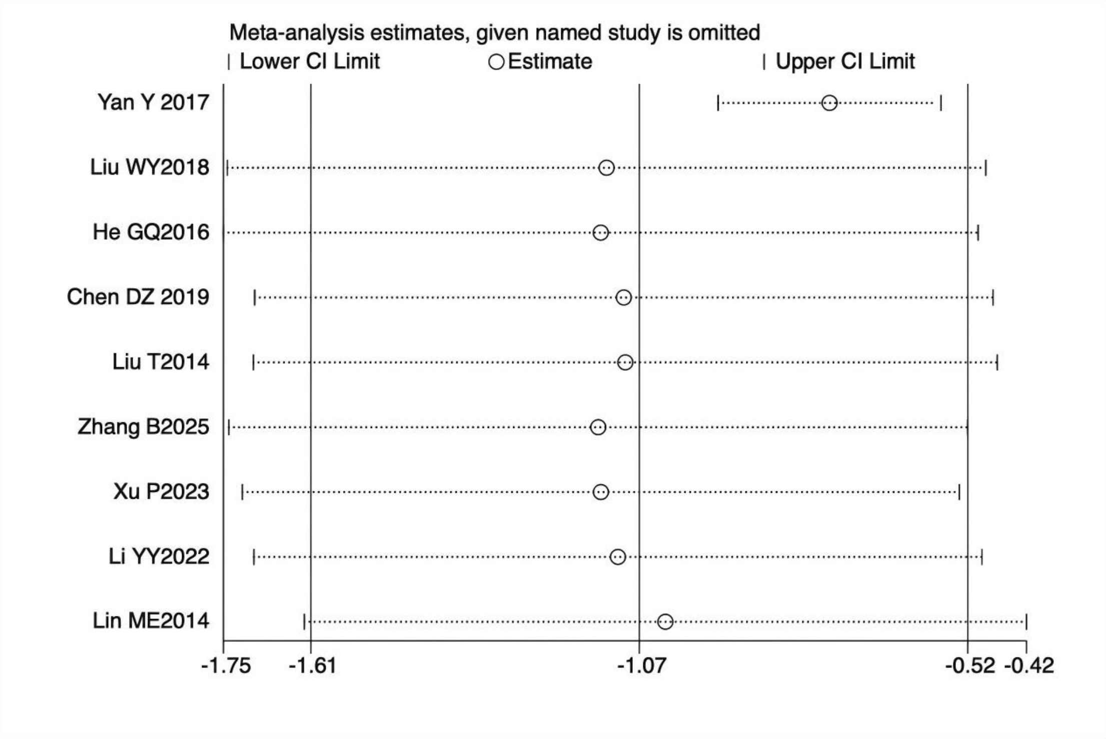
Meta-analysis estimates, given named study is omitted
Lower CI Limit
Estimate
Upper CI Limit
Yan Y 2017
Liu WY2018
He GQ2016
Chen DZ 2019
Liu T2014
Zhang B2025
Xu P2023
Li YY2022
Lin ME2014
-1.75
-1.61
-1.07
-0.52
-0.42
